# An optically and biomechanically corneal tissue-matched dual-crosslinkable hydrogel for cell-free remodeling of the corneal stroma

**DOI:** 10.1016/j.bioactmat.2026.10.032

**Published:** 2026-10-31

**Authors:** Wei Wu, Shuo Huai, Chenhao Li, Ling Wang, Siqi Xie, Lei Lei, Xiaoying Liu, Jiaqing Wang, Xingyi Li

**Affiliations:** a National Engineering Research Center of Ophthalmology and Optometry, Zhejiang Key Laboratory of Ophthalmic Drug Discovery and Medical Device Research, Eye Hospital, Wenzhou Medical University, 270 Xueyuan Road, Wenzhou, 325027, China; b Department of Ophthalmology, Guizhou Provincial People's Hospital, 83 Zhongshan East Road, Guiyang, 550002, China

**Keywords:** *In situ* dual-crosslinkable hydrogel, Corneal adaptive, Corneal stromal remodeling, Infection

## Abstract

Corneal trauma is a leading cause of visual impairment and blindness worldwide, yet corneal stromal repair and regeneration remain a significant challenge in ophthalmology because of its limited intrinsic repair capacity and fibrotic scar formation during healing process. Here we report an optically and biomechanically corneal tissue-matched *in situ* dual-crosslinkable hydrogel that enables cell-free corneal stromal remodeling. By integrating dynamic covalent crosslinking with static photocrosslinking strategy, the optimized hydrogel-based substitute achieved high optical transparency, a refractive index of approximately 1.35, and an elastic modulus of 83.8 ± 13.4 kPa, closely resembling the properties of the healthy human cornea. In a rabbit model of deep corneal defects, the optimized *in situ*-forming hydrogel adapted to the defect geometry, adhered stably to the wound bed, recapitulated the optical and biomechanical properties of the native cornea, and supported scarless corneal stromal remodeling. Incorporation of the antimicrobial peptide HHC36 further endowed the hydrogel with robust antibacterial activity without compromising its optical and biomechanical properties. In a rabbit model of *P. aeruginosa*–infected deep corneal defects, the HHC36-loaded *in situ*-forming hydrogel effectively mitigated clinical signs of infection and substantially promoted corneal stromal remodeling within 30 days while reducing fibrosis. Overall, this optically and biomechanically corneal tissue-matched *in situ* dual-crosslinkable hydrogel might be a promising substitute for corneal stromal remodeling.

## Highlights

• Dual-crosslinkable hydrogel matches native corneal optics and biomechanics.
• *In situ* hydrogel enables cell-free remodeling of deep corneal stroma.
• Dynamic and photo-crosslinking improve tissue integration and stability.
• HHC36-loaded hydrogel eradicates infection and reduces corneal fibrosis.
• Functional corneal remodeling is achieved in infected rabbit defects.

## Graphical abstract

An optically and biomechanically corneal tissue-matched dual-crosslinkable GelMA/OHAMA hydrogel loaded with antimicrobial peptide HHC36 provides simultaneous antibacterial, anti-inflammatory, and anti-fibrotic activities, enabling functional reconstruction of infected corneal stromal defects.

**Figure:**
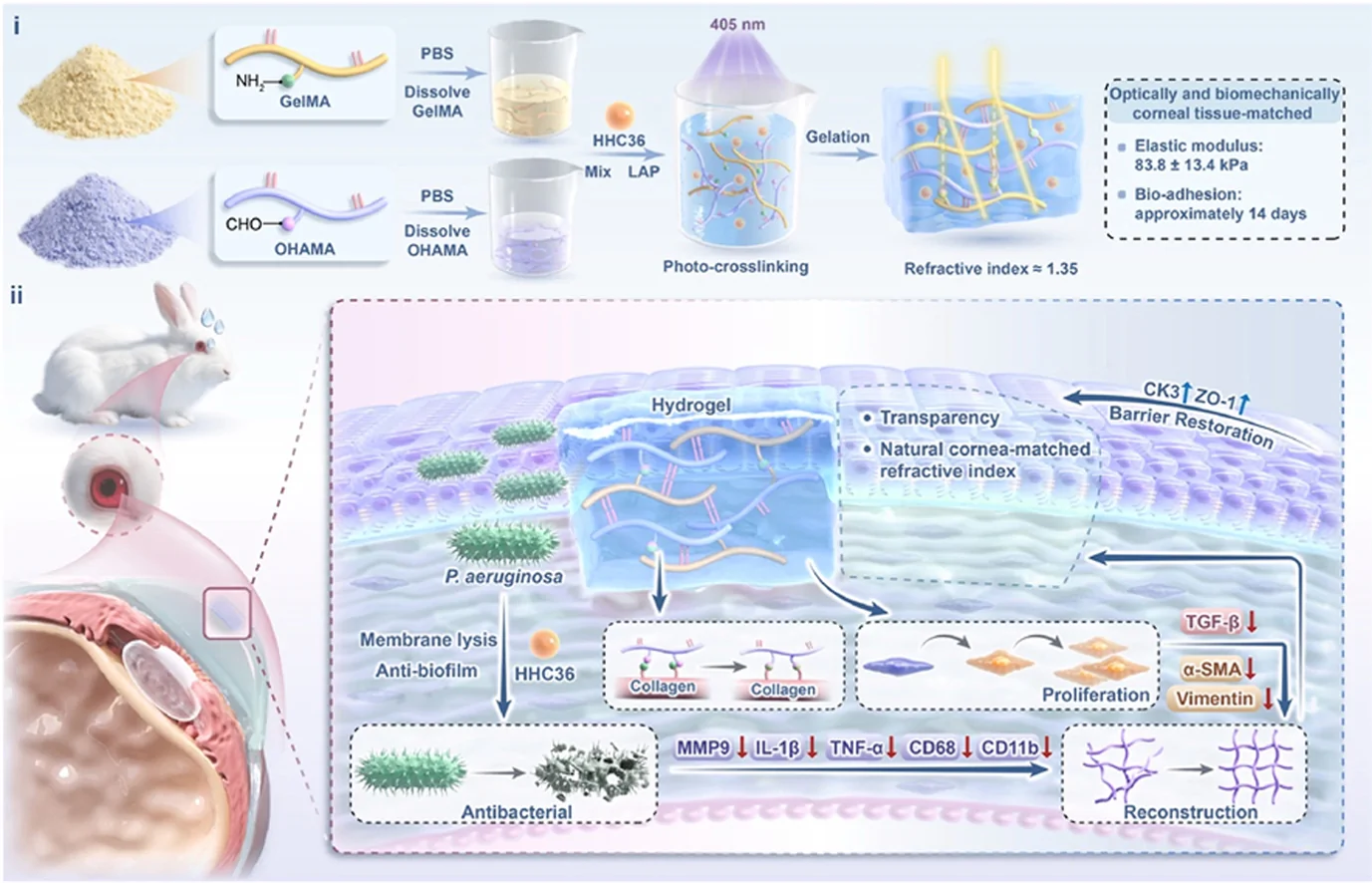

## 1 Introduction

Moderate to severe corneal injuries and infections can cause irreversible damage and are the leading causes of visual dysfunction and blindness [1], [2]. These injuries typically involve damage extending into the corneal stroma and, in more severe cases, the endothelium. The clinical intervention of moderate and severe corneal trauma remains a challenge in ophthalmology, primarily due to stromal fibrosis during the healing process, which leads to corneal scarring and haze and consequently markedly reduces corneal transparency and refractive index [3], [4], [5]. Currently, drug therapy, corneal transplantation, and stem cell therapy constitute the main clinical strategies for promoting corneal wound healing [6], [7], [8]. Although certain drug therapies, including anti-inflammatory agents and growth factors, can facilitate corneal wound repair, achieving orderly tissue architecture and preventing scar formation remain major challenges [4]. Cell therapy, particularly stem cell therapy, holds great promise for enhancing corneal regeneration; however, it remains largely at the preclinical stage and is limited by the availability of suitable stem cell sources [7]. In contrast, corneal transplantation remains the definitive treatment for severe corneal trauma, but its application is substantially constrained by the shortage of donor corneas [9]. Therefore, the development of novel corneal substitutes to overcome the limitations of current therapeutic approaches represents a critical and unmet medical need.

Over the past two decades, substantial progress has been achieved in the development and optimization of hydrogel-based substitutes for the management of moderate to severe corneal trauma [10], [11], [12], [13]. Both natural and synthetic hydrogels have been investigated for applications ranging from stromal repair to full-thickness corneal regeneration [6], [14], [15]. For example, polyethylene glycol (PEG) hydrogel-based substitutes, including ReSure® and OcuSeal®, have been clinically approved for the treatment of corneal incisions and small-diameter of corneal perforations [16], [17]. GelCORE, a recently developed hydrogel formed by photo-crosslinking GelMA, has demonstrated excellent corneal adhesion and effective stromal regeneration [18]. More recently, David Myung and colleagues reported a composite hyaluronan-collagen (HA-Col) hydrogel, crosslinked via either photochemistry or bio-orthogonal chemistry, as an alternative to corneal transplantation. Notably, HA-Col gels crosslinked through bio-orthogonal chemistry resulted in more rapid and optically favorable wound healing compared with those crosslinked by photochemical methods [1]. Despite these significant advances, the mismatch between hydrogel properties, such as optical performance, and biomechanical characteristics, and those of native corneal tissue often leads to suboptimal corneal regeneration outcomes. Therefore, developing hydrogel-based substitutes that closely recapitulate the optical and biomechanical properties of native corneal tissue remains a critical and unresolved challenge.

An ideal hydrogel-based corneal substitute should be transparent to visible light and provide appropriate refractive power. Meanwhile, it should possess biomechanical properties comparable to those of the native cornea to maintain corneal curvature and withstand intraocular pressure (IOP) [19], [20], [21]. Recently, we reported the development of an *in situ*-forming hydrogel substitute based on a photocrosslinking strategy, which was suitable for suture-free corneal repair. In this approach, an aqueous solution of hydrogel precursors was introduced into a surgically created corneal stromal defect, where it was transformed into a hydrogel scaffold upon photocrosslinking. The resulting scaffold adhered to the underlying stroma and promoted corneal stromal regeneration [22]. Such static hydrogel possesses stable network structures that provide mechanical strength and structural integrity; however, they often fail to match the dynamic biomechanical nature of the native human cornea. Therefore, dynamic hydrogels that mimic the adaptive optical and biomechanical characteristics of the native cornea offer promising opportunities for corneal stromal regeneration [23]. By integrating dynamic covalent crosslinking with static photocrosslinking, we engineered an optically and biomechanically corneal tissue-matched *in situ* dual-crosslinkable hydrogel based on oxidized hyaluronic acid methacryloyl (OHAMA) and gelatin methacryloyl (GelMA) for cell-free corneal stromal remodeling. Incorporation of a cationic antimicrobial peptide (KRWWKWWRR; HHC36) further imparted the hydrogel with robust antibacterial activity without influencing its optical and biomechanical properties. Using rabbit models of deep corneal defects and *P. aeruginosa*–infected deep corneal defects, we demonstrated that the proposed optically and biomechanically corneal tissue-matched *in situ* dual-crosslinkable hydrogel enables cell-free stromal remodeling while reducing fibrosis (Scheme 1).

**Scheme 1:**
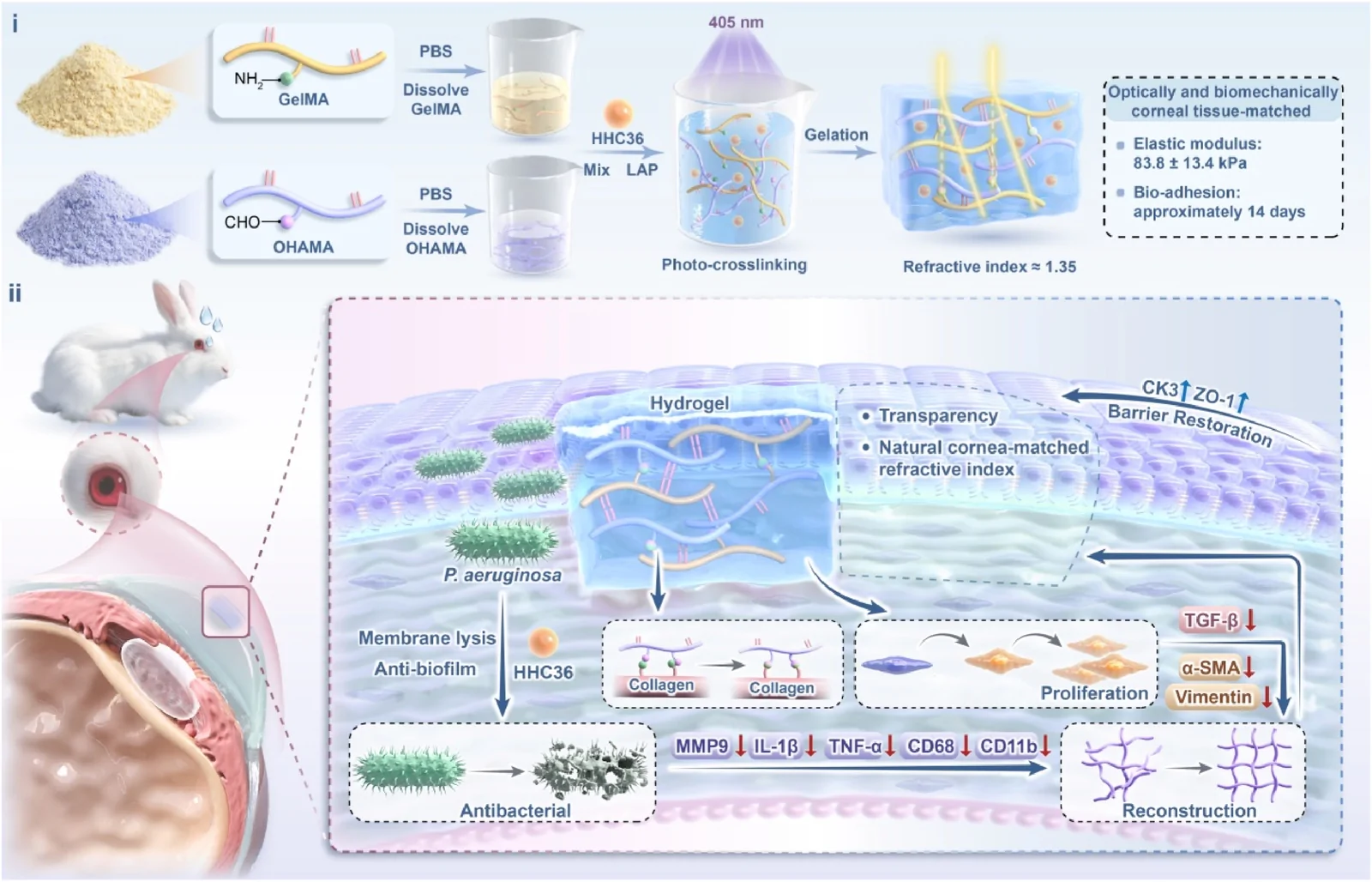
Schematic illustration of an optically transparent and biomechanically tissue-matched dual-crosslinkable hydrogel enables cell-free corneal stromal remodeling in rabbit models of deep corneal defect and *P. aeruginosa*–infected deep corneal defect.

## 2 Results

### 2.1 Rational design, preparation and characterization of hydrogel-based substitutes

Corneal stromal substitutes should serve not merely as passive fillers, but as intelligent structural supports capable of interacting with the surrounding tissue microenvironment and mimicking the corneal physiological functions [24], [25], [26], [27]. Here we synthesized oxidized hyaluronic acid methacryloyl (OHAMA) and integrated dynamic covalent crosslinking with static photocrosslinking to develop an optically and biomechanically corneal tissue-matched gelatin methacryloyl (GelMA)–OHAMA *in situ*-forming hydrogel for cell-free corneal stromal remodeling. GelMA, which contains abundant cell-adhesive motifs, supports corneal epithelial and stromal cell adhesion, migration, and organized tissue regeneration. Hyaluronic acid (HA), a native component of the corneal extracellular matrix, provides excellent tissue compatibility, hydration capacity, and support for stromal remodeling. Through dynamic Schiff-base interactions and photocrosslinking, GelMA and OHAMA form a dual-crosslinked network that combines optical transparency, adaptive mechanics, controlled degradability, and compatibility with the corneal repair microenvironment, making it well suited for *in situ* corneal defect repair. To prepare OHAMA, HA was first oxidized with sodium periodate (NaIO_4_) to introduce aldehyde groups and was subsequently methacrylated with methacrylic anhydride (Scheme S1). The identity of OHAMA was identified by ^1^H NMR and the weight-average molecular weight (M_w_) of OHAMA was 442,675 Da as analyzed by gel permeation chromatography (GPC) (Figure S1, and Figure S2). Schiff-base reactions between aldehyde groups in OHAMA and amino groups in GelMA generated a dynamic, low-viscosity network suitable for injection. After injection into the defect, residual aldehyde groups formed covalent bonds with amino groups in the corneal tissue, strengthening host–hydrogel integration and bioadhesion. *In situ* photocrosslinking under 405 nm visible-light irradiation then converted the dynamic, low-viscosity matrix into a dual-crosslinked hydrogel for cell-free stromal remodeling.

Fourier transform infrared (FTIR) and Raman spectroscopy were employed to characterize the chemical structures of HA, OHAMA, GelMA, the hydrogel precursor solution, and the photocrosslinked hydrogel. In the FTIR spectra (Fig. S3a), OHAMA exhibited characteristic absorption bands at approximately 1630 and 1730 cm^−1^, corresponding to methacryloyl C=C stretching and ester C=O stretching, respectively, confirming the introduction of methacryloyl groups. The aldehyde C=O absorption near 1720 cm^−1^ may overlap with the ester carbonyl band at 1730 cm^−1^. Due to the spectral overlap between imine C=N and methacryloyl C=C stretching vibrations, the hydrogel spectra were further compared before and after photocrosslinking. The precursor solution displayed an overlapping absorption band near 1650 cm^−1^, attributed to C=N and C=C stretching vibrations. Following photopolymerization, the intensity of this band decreased markedly, indicating substantial consumption of methacryloyl double bonds, while the residual absorption was consistent with the presence of imine linkages. Raman spectroscopy revealed a band at approximately 1652 cm^−1^, providing complementary evidence for the proposed chemical structure (Fig. S3b). Collectively, these spectroscopic results support the formation of a dual-crosslinked hydrogel network through Schiff base linkages (C=N) and free-radical polymerization of methacryloyl groups (C=C).

To adapt the optical and biomechanical properties of the native cornea, the properties of hydrogel was optimized. As shown in Fig. 1a, the hydrogel rapidly formed upon photo-crosslinking at 405 nm. GelMA/OHAMA mixtures with varying compositions yielded transparent hydrogels (Fig. 1b). The formulations containing 100 mg/mL GelMA and 5, 10, or 20 mg/mL OHAMA were designated G_100_OH_5_, G_100_OH_10_, and G_100_OH_20_, respectively. All hydrogels exhibited optical transmittance above 80% at 500–800 nm, which was higher than that of the native cornea (78%) [28]. Notably, optical transmittance declined modestly with increasing OHAMA content, probably owing to enhanced light scattering caused by higher crosslinking density and reduced network homogeneity (Fig. 1c). The refractive indices of GelMA, G_100_OH_5_, G_100_OH_10_, and G_100_OH_20_ hydrogel were 1.357 ± 0.0002, 1.351 ± 0.001, 1.344 ± 0.002, and 1.341 ± 0.002, respectively (Fig. 1d), which were comparable to that of the native cornea (approximately 1.376). It is worth noting that the refractive indices of hydrogel gradually decreased with an increasement of OHAMA content. Scanning electron microscopy (SEM) images showed that all hydrogels exhibited similar three-dimensional (3D) network structures with uniformly distributed, nearly circular pores (Fig. 1e). This highly porous and interconnected architecture facilitates the diffusion of water, nutrients, and metabolic waste, providing a favorable microenvironment for cell proliferation. With increasing OHAMA content, the hydrogel exhibited a denser pore structure, characterized by smaller pore sizes and lower porosity (Fig. S4), indicating enhanced crosslinking density of the hydrogel network. As shown in Fig. 1f and g, all hydrogels exhibited water contact angles below 90°, confirming their hydrophilic nature. GelMA showed the highest contact angle of 72.37 ± 4.36°, whereas the values gradually decreased to 72.14 ± 3.66°, 63.57 ± 3.87°, and 55.58 ± 2.71° for G_100_OH_5_, G_100_OH_10_, and G_100_OH_20_, respectively, implying enhanced hydrophilicity. This enhancement was primarily attributed to the hydrophilic nature of HA, which introduced additional hydrophilic groups onto the hydrogel surface.

**Fig. 1:**
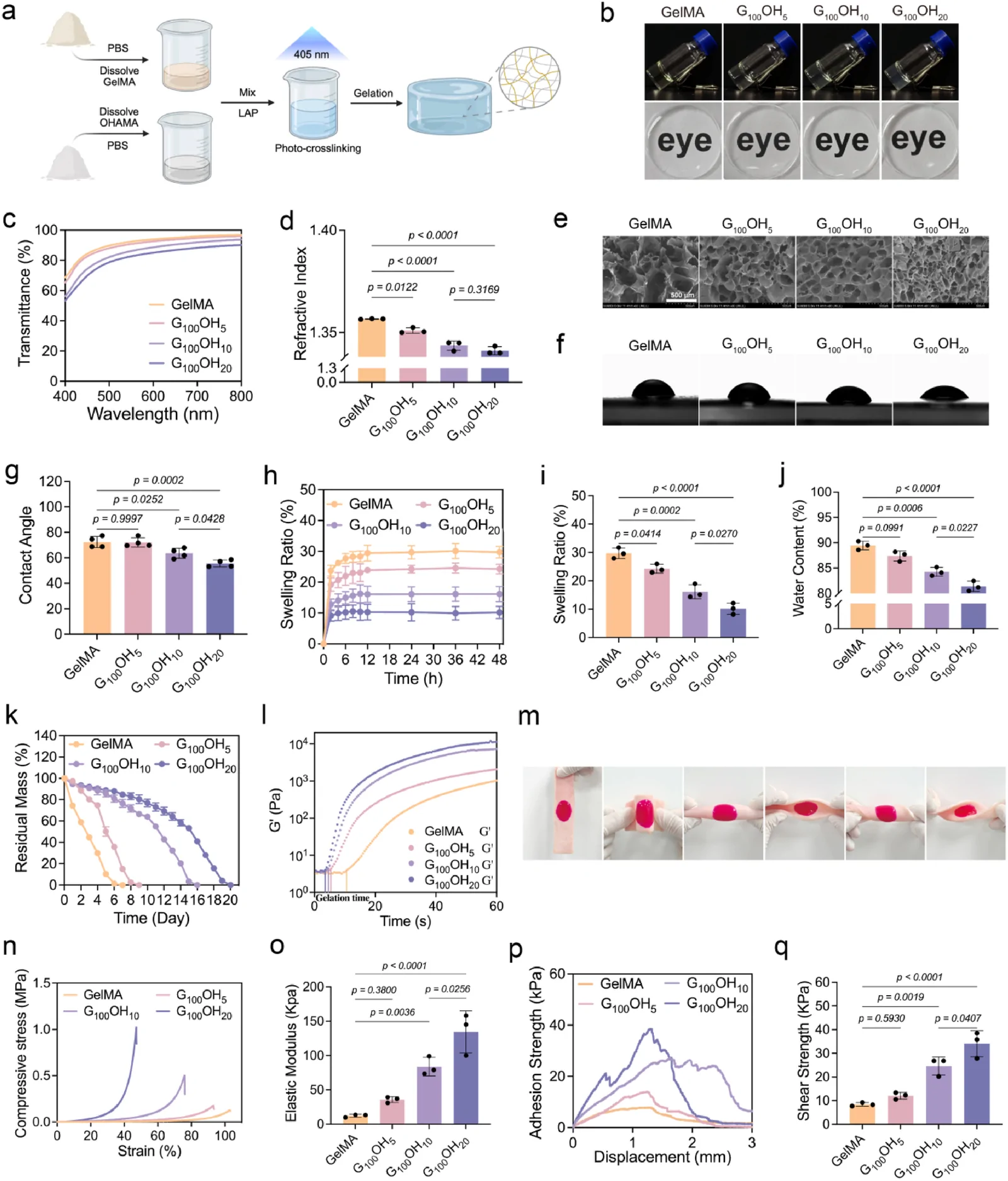
**Fabrication and characterization of various hydrogels.** (a) Schematic illustration of photo-crosslinked hydrogel preparation. (b) Inverted-vial test and representative photograph showing the optical transparency of various hydrogels. (c) Transmittance spectra of various hydrogels in the wavelength range of 400–800 nm. (d) Quantification of the refractive index of various hydrogels. (e) SEM image showing the porous microstructure of various hydrogels. Scale bar: 500 μm. (f) Representative contact angle images of various hydrogels. (g) Quantification of the contact angles of various hydrogels. (h) Swelling kinetics of various hydrogels. (i) Quantification of the swelling ratio of various hydrogels after 48 h incubation. (j) Quantification of the water content of various hydrogels. (k) Degradation profiles of various hydrogels in PBS containing collagenase type II and hyaluronidase. (l) Time-dependent storage modulus of various hydrogels. (m) Representative photographs showing hydrogel adhesion to porcine skin under different conditions. (n) Compressive stress–strain curves of various hydrogels. (o) Quantification of the elastic modulus of various hydrogels. (p) Representative adhesion force curves of various hydrogels. (q) Quantification of the shear force of various hydrogels derived from the corresponding adhesion force curves. Data are presented as mean ± SD [(d, h–k, o–q) n = 3 independent samples; (g) n = 4 independent samples] and analyzed by one-way ANOVA with multiple comparisons.

GelMA, G_100_OH_5_, G_100_OH_10_, and G_100_OH_20_ hydrogels reached swelling equilibrium after 12 h, with swelling ratio of 29.73 ± 3.56%, 24.26 ± 2.37%, 16.14 ± 6.17%, and 10.17 ± 3.99% at 48 h, respectively, showing significant differences among groups (Fig. 1h and i). The swelling ratio decreased with increasing OHAMA content, likely due to the higher crosslinking density and denser network structure. This relatively low swelling capacity is advantageous for maintaining corneal curvature, reducing mechanical stress, preserving structural stability, and promoting integration with the corneal stroma [29]. The water contents of GelMA, G_100_OH_5_, G_100_OH_10_, and G_100_OH_20_ hydrogels were 89.46 ± 0.72%, 87.39 ± 0.98%, 84.26 ± 0.75%, and 81.39 ± 1.02%, respectively (Fig. 1j), comparable to that of the native human cornea, approximately 76–86%. Such a high content helps maintain optical transparency, mimic the hydration and biomechanical characteristics of the native cornea, and provide a favorable environment for cell proliferation. Enzymatic degradation assays using collagenase and hyaluronidase demonstrated that hydrogel degradation resistance increased with OHAMA content (Fig. 1k). Specifically, GelMA hydrogel degraded completely within approximately 6 days, whereas G_100_OH_20_ hydrogel persisted for approximately 20 days. This enhanced stability may result from the increased crosslinking sites and denser network formed at the higher OHAMA concentrations, which restricted enzyme penetration and diffusion.

The dynamic rheological properties of various hydrogels were assessed using a photorheometer by monitoring the storage modulus (G′) over time. As shown in Fig. 1l, the sharp increase in G′ value of system reflects the sol–gel transition, and the corresponding time was defined as the photo-induced gelation time. The gelation times of GelMA, G_100_OH_5_, G_100_OH_10_, and G_100_OH_20_ were 10, 5.5, 5, and 4.5 s, respectively, while their corresponding G′ values at 60 s were 1,020, 2,078, 7,288, and 11,328 Pa. Collectively, these results demonstrate that OHAMA incorporation accelerated photo-crosslinking, with increasing OHAMA content progressively shortening the gelation time and markedly enhancing hydrogel stiffness.

Subsequently, the hydrogels were labeled with rhodamine B, applied to porcine skin, and crosslinked upon exposure to 405 nm blue light. As shown in Fig. 1m, the hydrogels adhered firmly to the skin surface and remained stable under twisting, compression, and stretching, demonstrating excellent tissue adhesion. To further evaluate the adhesive performance of the hydrogels and assess their potential clinical applicability, adhesion tests were performed using porcine corneal tissue. As shown in Fig. S5, the burst pressures of GelMA, G_100_OH_5_, G_100_OH_10_, and G_100_OH_20_ were 74.50 ± 7.75, 81.00 ± 11.02, 99.75 ± 11.27, and 104.00 ± 9.83 mmHg, respectively. Given that normal intraocular pressure typically ranges from 10 to 20 mmHg, the burst pressures of all tested hydrogels substantially exceeded the physiological intraocular pressure range, indicating robust adhesion to the corneal tissue. The compressive properties of hydrogels are critical for maintaining structural integrity and long-term stability under intraocular pressure. As shown in Fig. 1n, GelMA exhibited a compressive strength of 0.13 MPa. OHAMA incorporation enhanced hydrogel rigidity and resistance to volumetric deformation, with compressive strength increasing with OHAMA content and reaching 1.03 MPa in the G_100_OH_20_ hydrogel. Similarly, the elastic modulus increased from 12.30 ± 2.00 kPa for GelMA hydrogel to 35.73 ± 4.24, 83.82 ± 13.43, and 134.40 ± 30.47 kPa for G_100_OH_5_, G_100_OH_10_, and G_100_OH_20_ hydrogel, respectively (Fig. 1o), approaching that of the native cornea (115.30 ± 13.60 kPa) [18]. Adhesion in a moist ocular environment is also essential for corneal stromal repair and remodeling. Fresh porcine skin was therefore used as a model tissue to evaluate hydrogel adhesion. As shown in Fig. 1p and q, GelMA hydrogel showed relatively low shear adhesion strength of 8.42 ± 0.74 kPa. After OHAMA incorporation, the values increased to 12.06 ± 1.43, 24.63 ± 3.82, and 34.07 ± 5.53 kPa for G_100_OH_5_, G_100_OH_10_, and G_100_OH_20_ hydrogel, respectively. The enhanced adhesion is likely attributable to Schiff-base formation and covalent bonding between the hydrogel network and the tissue surface. Altogether, these results suggest that the biomechanical and bioadhesive properties of the hydrogel can be precisely tuned by varying the OHAMA content to match those of native corneal tissue.

### 2.2 *In vitro* biocompatibility and cell migration ability of hydrogel-based substitutes

We next evaluated the cytotoxicity of various hydrogels toward L929 and HCEC cells using the CCK-8 assay. As shown in Fig. 2a and b, the OD values of all tested hydrogel extracts were comparable to those of the Control group and increased over time, indicating no obvious cytotoxicity. The cytocompatibility of the hydrogels was then evaluated using a two-dimensional (2D) cell culture assay. As shown in Fig. 2c–d and Fig. S6a–b, cell viability remained above 90% in all groups throughout the culture period, accompanied by a progressive increase in cell number. However, G_100_OH_20_ exhibited slightly reduced cell proliferation on day 5, potentially due to the increased crosslinking density and matrix stiffness associated with higher OHAMA concentrations, which may restrict cell spreading [30]. To further recapitulate the *in vivo* three-dimensional (3D) microenvironment, cells were encapsulated within the hydrogels, and their viability and proliferation were assessed on days 1, 3, and 5. As shown in Fig. 2e–f and Fig. S6c–d, cell viability remained above 60% in all groups, although a significant decrease was observed in G_100_OH_20_ on day 5. This reduction may be attributed to the denser polymer network, which could limit oxygen and nutrient diffusion while impeding metabolic waste removal [31]. Cell counts showed no significant differences among the groups on days 1 and 3. By day 5, G_100_OH_10_ maintained a relatively high cell number, suggesting a more favorable microenvironment for cell proliferation. In contrast, the lower crosslinking densities and weaker mechanical properties of GelMA and G_100_OH_5_ may compromise structural stability, thereby limiting cellular proliferation [32]. Collectively, these results highlight the importance of balancing crosslinking density, mechanical stability, and mass transport to create a supportive hydrogel microenvironment for cell survival and proliferation. A scratch assay was performed to evaluate the ability of the hydrogels to promote HCEC migration (Fig. 2g and h). At 24 h, the migration areas of the Control, G_100_OH_5_, G_100_OH_10_, and G_100_OH_20_ hydrogels were 43.32 ± 2.25%, 44.42 ± 3.80%, 48.92 ± 2.81%, and 44.99 ± 3.72%, respectively. At 36 h, the corresponding values increased to 78.97 ± 5.25%, 79.96 ± 6.05%, 87.80 ± 4.41%, and 82.64 ± 5.50%, respectively. These results demonstrated that the hydrogels promoted HCEC migration within 36 h, with G_100_OH_10_ hydrogel showing the strongest migration-supporting effect. Overall, the GelMA and G_100_OH_5_ groups exhibited insufficient mechanical stability, rapid degradation and structural collapse, together with poor cell spreading and aggregation, whereas G_100_OH_20_ showed high viscosity and limited adaptability, with excessive stiffness that may hinder cell proliferation. In contrast, G_100_OH_10_ provided the most favorable balance of optical transparency, cornea-matched mechanical properties, cytocompatibility, degradation behavior, tissue adhesion, and support for cell migration and growth, and was therefore selected for subsequent *in vivo* studies.

**Fig. 2:**
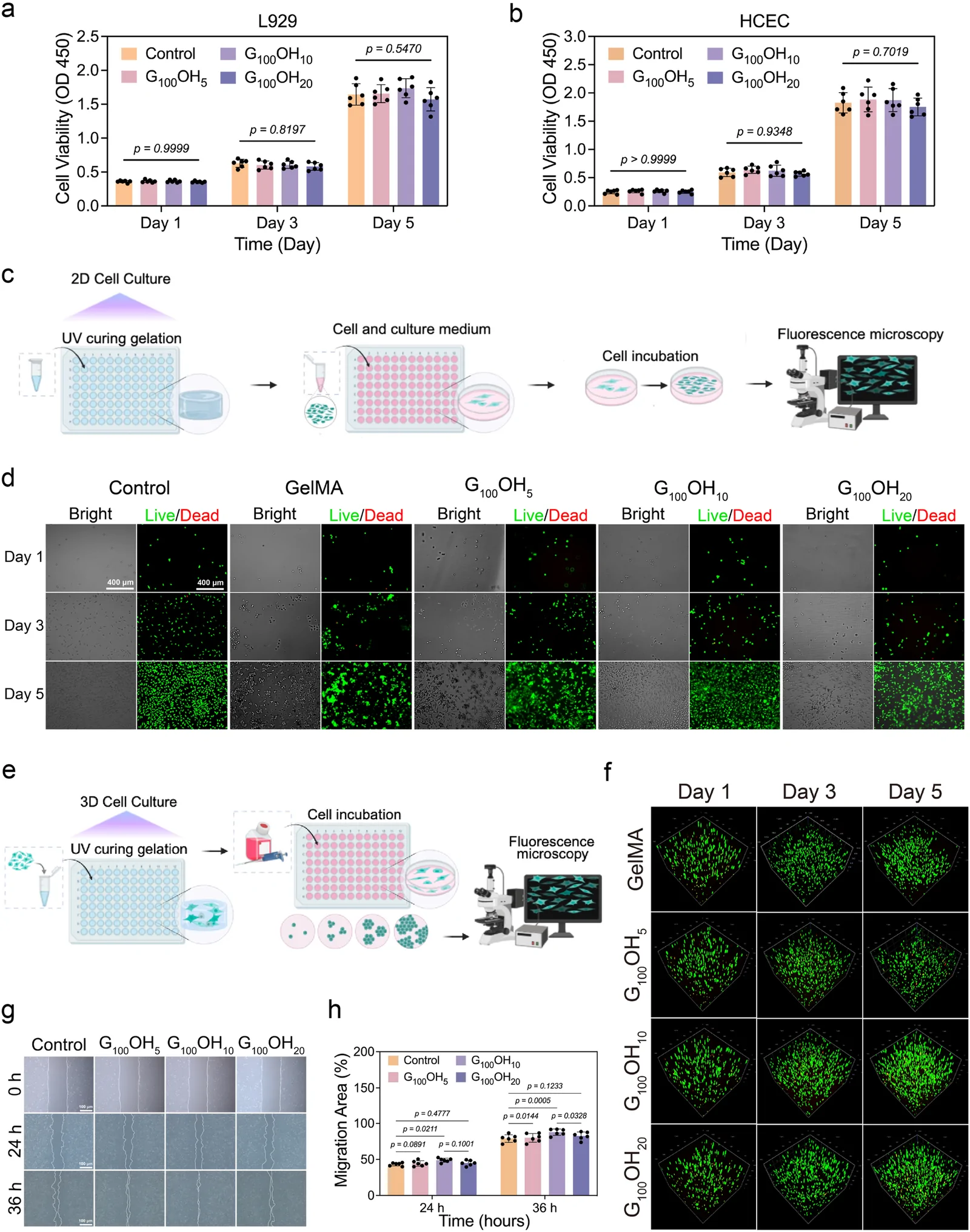
***In vitro* cellular effect of various hydrogels.** Cell viability of (a) L929 and (b) HCEC cells treated with different hydrogel extracts, as assessed by CCK-8 assay. (c) Schematic illustration of the 2D cell culture and evaluation procedure. (d) Live/dead staining images of L929 cells cultured with various hydrogels. Live cells are shown in green, and dead cells are shown in red. Scale bar: 400 μm. (e) Schematic illustration of the 3D cell culture and evaluation procedure. (f) Representative images of L929 cells after 3D incubation with various hydrogels. (g) Representative scratch assay images of HCEC cells treated with different hydrogel extracts. Scale bar: 100 μm. (h) Quantification of scratch wound closure in HCEC cells. Data are presented as mean ± SD [(a, b) n = 6 independent samples, one-way ANOVA with multiple comparisons; (h) n = 6 independent samples, two-way ANOVA with multiple comparisons].

### 2.3 G_100_OH_10_ hydrogel promoting the corneal stromal remodeling in a rabbit model of deep corneal defect

To evaluate the potential of the dual-crosslinkable *in situ* hydrogel (G_100_OH_10_ hydrogel) for cell-free corneal stromal remodeling, a rabbit model of deep corneal defect was established by anterior lamellar keratectomy using surgical trephination (Fig. 3a). As shown in Fig. 3b, slit-lamp examination revealed that G_100_OH_10_ hydrogel adhered firmly to the corneal stroma and maintained a smooth, transparent surface at postoperative day 0. The absence of corneal edema, conjunctival congestion or edema, increased ocular secretion, and anterior chamber flare throughout follow-up suggests favorable ocular biocompatibility. G_100_OH_10_ hydrogel-treated group achieved complete corneal epithelialization by postoperative day 7, whereas epithelial defects persisted in the Model group. By day 90, G_100_OH_10_ hydrogel-treated group showed markedly reduced corneal opacity relative to the Model group. Anterior segment optical coherence tomography (AS-OCT) imaging showed that the G_100_OH_10_ hydrogel effectively filled the corneal defect and conformed well to the native corneal curvature at day 7 (Fig. 3c), while remaining firmly adhered to the corneal tissue at day 14 (Fig. S7). By day 90, both Model and G_100_OH_10_ hydrogel groups exhibited varying degrees of stromal scarring. The Model group showed thickened, irregular hyperreflective regions indicative of severe scar formation, whereas G_100_OH_10_ hydrogel-treated corneas exhibited only mild stromal hyperreflectivity and near-native morphology, suggesting that the G_100_OH_10_ hydrogel effectively reduced scarring during corneal stromal remodeling.

**Fig. 3:**
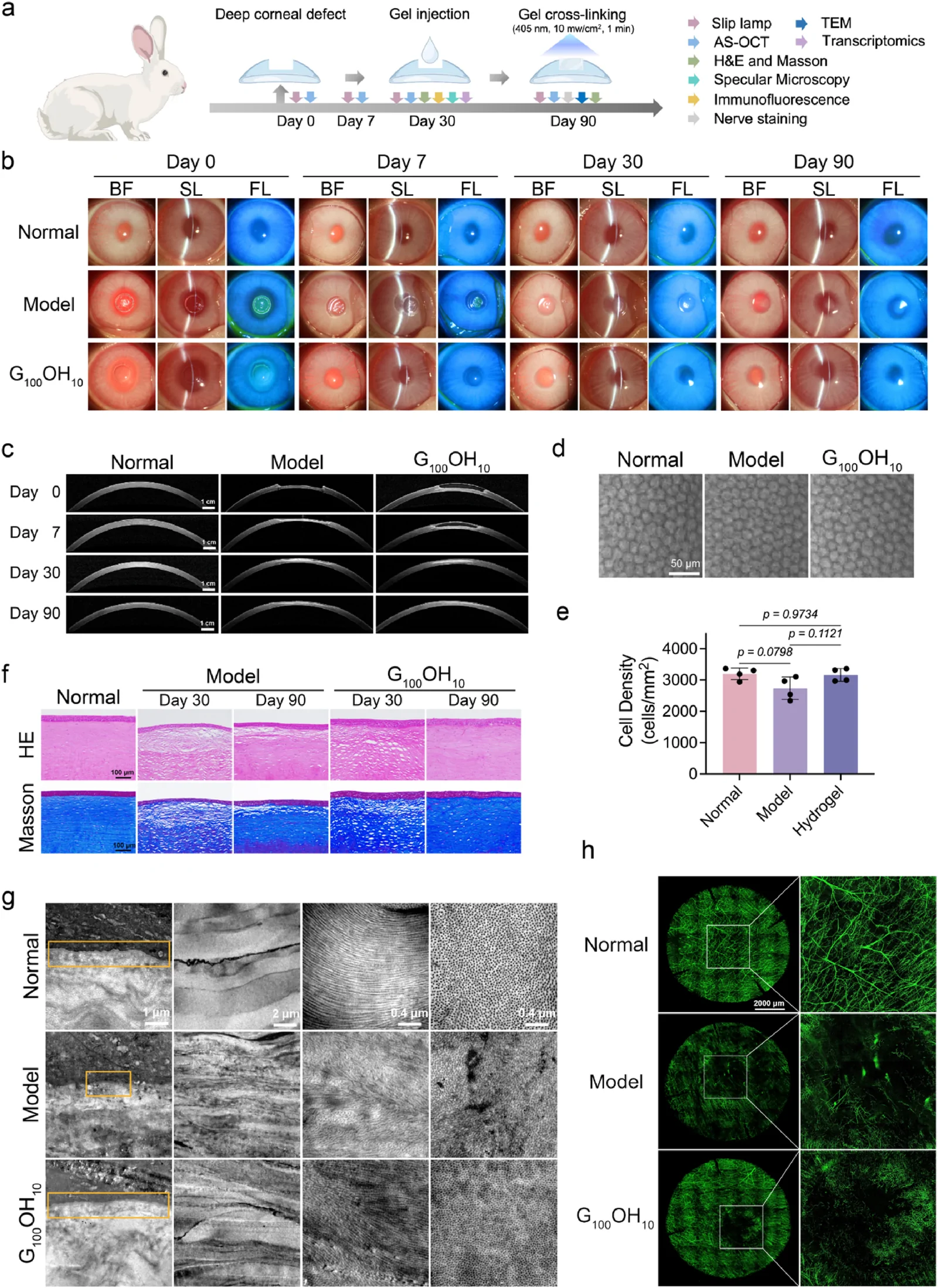
***In vivo* performance of G_100_OH_10_ hydrogel in a rabbit model of deep corneal stromal defects.** (a) Schematic illustration of model establishment, treatment protocol, and outcome assessment. The Normal group was rabbit without any manipulation. In the Model group, a localized corneal epithelial and stromal defect with a depth of approximately half of the full corneal thickness was created using a 3-mm corneal trephine. In the Hydrogel group, the same corneal defect model was established and treated with G_100_OH_10_ hydrogel alone. (b) Representative bright-field (BF), slit-lamp (SL), and fluorescein staining (FL) images of rabbit eyes from different groups. (c) Representative AS-OCT images of rabbit corneas from different groups. (d) Representative corneal specular microscopy images of the corneal endothelium from different groups at day 30. (e) Quantification of corneal endothelial cell density from different groups at day 30. (f) H&E and Masson staining images of rabbit corneal tissues from different groups at day 30, and 90. (g) TEM images of the anterior corneal stroma on postoperative day 90. The yellow box indicates the basement membrane. Images are shown from left to right as the epithelial–stromal interface (scale bar: 1 μm), anterior stroma (scale bar: 2 μm), and two sectional views of anterior stromal collagen fibers (scale bar: 0.4 μm). (h) Immunofluorescence staining of corneal nerves labeled with βIII-tubulin from different groups on postoperative day 90, with corresponding magnified images. Scale bar: 2000 μm. Data are presented as mean ± SD (n = 4 independent samples) and analyzed by one-way ANOVA with multiple comparisons.

Moderate to severe corneal trauma is typically associated with stromal fibrotic remodeling and corneal endothelial injury [33], [34]. We therefore used specular microscopy to assess corneal endothelial status across the different groups. As shown in Fig. 3d and e, neither the Model group nor the Hydrogel-treated group exhibited obvious changes in endothelial cell density or morphology compared with the Normal group. These finding indicated that G_100_OH_10_ hydrogel implantation did not adversely affect the structure and morphology of the corneal endothelium. Masson's trichrome staining further showed marked differences in stromal collagen organization between the Model and G_100_OH_10_ hydrogel groups (Fig. 3f). On postoperative day 30, collagen fibers in the Model group were loosely and irregularly arranged, forming a disorganized stromal network accompanied by clustered myofibers. In contrast, the G_100_OH_10_ hydrogel group exhibited a more compact and ordered collagen architecture, although the stromal organization remained relatively loose at this stage. By day 90, the Model group exhibited an unstratified squamous corneal epithelium, accompanied by thickened, disordered and randomly oriented stromal collagen fibers. Conversely, the G_100_OH_10_ hydrogel group showed remodeling of a stratified squamous corneal epithelium resembling the normal corneal epithelium, together with more regularly aligned stromal collagen fibers that approached a tightly packed, parallel lamellar organization. Overall, the G_100_OH_10_ hydrogel implantation effectively promoted orderly stromal collagen remodeling and attenuated pathological fibrosis, which is critical for restoring corneal transparency.

In contrast, slit-lamp examination (Fig. S8a) showed that GelMA effectively filled and covered the corneal defect immediately after implantation; however, matrix scar formation became evident by day 30. This outcome suggests that, despite adequate initial wound coverage, the relatively low mechanical strength and rapid degradation of GelMA were insufficient to sustain defect stability during the early repair phase. The G_100_OH_20_ group also exhibited satisfactory early wound sealing, but pronounced scar-like matrix formation was observed at day 30. This may be attributed to its relatively high modulus and mechanical mismatch with the native corneal stroma, which likely impaired stromal cell migration and tissue remodeling during later stages of repair. Consistent with these observations, AS-OCT imaging (Fig. S8b) showed that GelMA was largely degraded by day 7, resulting in inadequate structural support, incomplete maintenance of defect filling, and irregular stromal remodeling. In contrast, the excessive mechanical strength of G_100_OH_20_ likely restricted orderly stromal cell infiltration. Although a small amount of hydrogel remained at day 30, the newly formed stromal tissue was thick and irregular. H&E staining (Fig. S8c) further confirmed these findings. In the GelMA group, premature scaffold degradation prolonged defect persistence and was associated with markedly disorganized collagen deposition. In the G_100_OH_20_ group, mild inflammatory cell infiltration was observed, while the high material rigidity appeared to disrupt collagen organization and limit stromal cell spreading, migration, and ordered extracellular matrix deposition. Collectively, these results highlight the importance of balancing scaffold stability, degradation kinetics, and mechanical compatibility with the native cornea. The dual-crosslinking architecture and biomechanical properties of G_100_OH_10_ appear to provide a more favorable microenvironment for corneal stromal remodeling by maintaining sufficient early structural support while permitting progressive cell infiltration and organized matrix remodeling. The divergent cellular responses likely reflect differences in spatial localization and microenvironment. Epithelial cells at the hydrogel interface benefit from greater oxygen and nutrient availability, supporting migration, proliferation, and barrier restoration. In contrast, stromal cells within the hydrogel experience sustained mechanical cues that may promote matrix remodeling while limiting myofibroblast activation and fibrosis. GelMA provides adhesive motifs that support epithelial attachment and migration, whereas OHAMA-derived hyaluronic acid may help maintain a quiescent stromal phenotype. Together, these spatial and compositional cues enable layer-specific regulation within a homogeneous hydrogel. Further studies are needed to clarify the underlying molecular mechanisms.

On postoperative day 90, we used the transmission electron microscopy (TEM) to assess stromal ultrastructure repair across the different groups. As shown in Fig. 3g, the normal cornea exhibited an intact basement membrane and well-organized stromal collagen lamellae with clearly defined interlamellar boundaries. Collagen fibrils were highly ordered and parallel, with uniform diameters and a homogeneous distribution. By contrast, the Model group showed extensive disruption of the basement membrane and disorganized stromal collagen lamellae with indistinct interlamellar boundaries, accompanied by irregularly aligned collagen fibrils with heterogeneous diameters, implying the fibrotic remodeling after injury. Notably, G_100_OH_10_ hydrogel group showed a more continuous basement membrane and markedly improved organization of stromal collagen lamellae. Parallel alignment of collagen fibrils was partially restored, accompanied by improved diameter uniformity and increased fibril density. Based on this result, the G_100_OH_10_ hydrogel implantation supports ultrastructural stromal repair through orderly collagen remodeling.

Given the dense innervation of the cornea, delayed nerve regeneration after injury may lead to neurotrophic keratopathy and indirectly compromise visual acuity [35], [36]. TUBB3 staining assay was utilized to evaluate corneal nerve repair at day 90 (Fig. 3h). The normal cornea exhibited a dense, intact, and well-organized neural network, whereas the defect area of Model group showed pronounced nerve loss, extensive nerve fiber disruption, and markedly reduced nerve-ending density. Following G_100_OH_10_ hydrogel implantation, the cornea showed increased nerve-ending signals and partial restoration of the neural network within the defect area compared with the Model group. These results demonstrated that G_100_OH_10_ hydrogel implantation was able to promote the repair of injured corneal nerves, although the regenerated neural network did not fully recapitulate the structural integrity and organization observed in normal corneal tissue.

To elucidate the possible molecular mechanism of G_100_OH_10_ hydrogel implantation on the promotion of corneal stromal remodeling with reduced scarring, transcriptomic analysis of corneal tissue from Hydrogel and Model groups was conducted on day 90. A cluster heatmap of differentially expressed genes involved in ECM synthesis, remodeling, and fibrosis was generated to compare the Model and Hydrogel groups (Fig. 4a). The Model group showed increased expression of *TGFB2*, *LTBP4*, *PDGFA*, and *COL10A1* genes associated with matrix degradation and fibrosis progression, indicating persistent dysregulation of ECM remodeling after injury. On the other hand, the Hydrogel group exhibited increased expression of genes including *LIMS2*, *ZYX*, *KRT4*, *KRT7*, *PAMR1*, and *THBS4*, which are primarily involved in collagen synthesis and growth-factor-mediated tissue regeneration. KEGG enrichment analysis further revealed that downregulated genes in the Hydrogel group were significantly enriched in fibrosis- and repair-related pathways, including ECM–receptor interaction and TGF-β signaling pathways (Fig. 4b). Additionally, GO enrichment analysis showed that these downregulated genes were mainly associated with biological processes such as cell adhesion, extracellular matrix organization, collagen metabolic processes, receptor-mediated signaling, negative regulation of cell proliferation, and cellular response to hypoxia (Fig. 4c). All these findings indicated that G_100_OH_10_ hydrogel implantation supported corneal stromal remodeling while reducing scar formation, partly through the suppression of TGF-β-mediated pathological ECM remodeling.

**Fig. 4:**
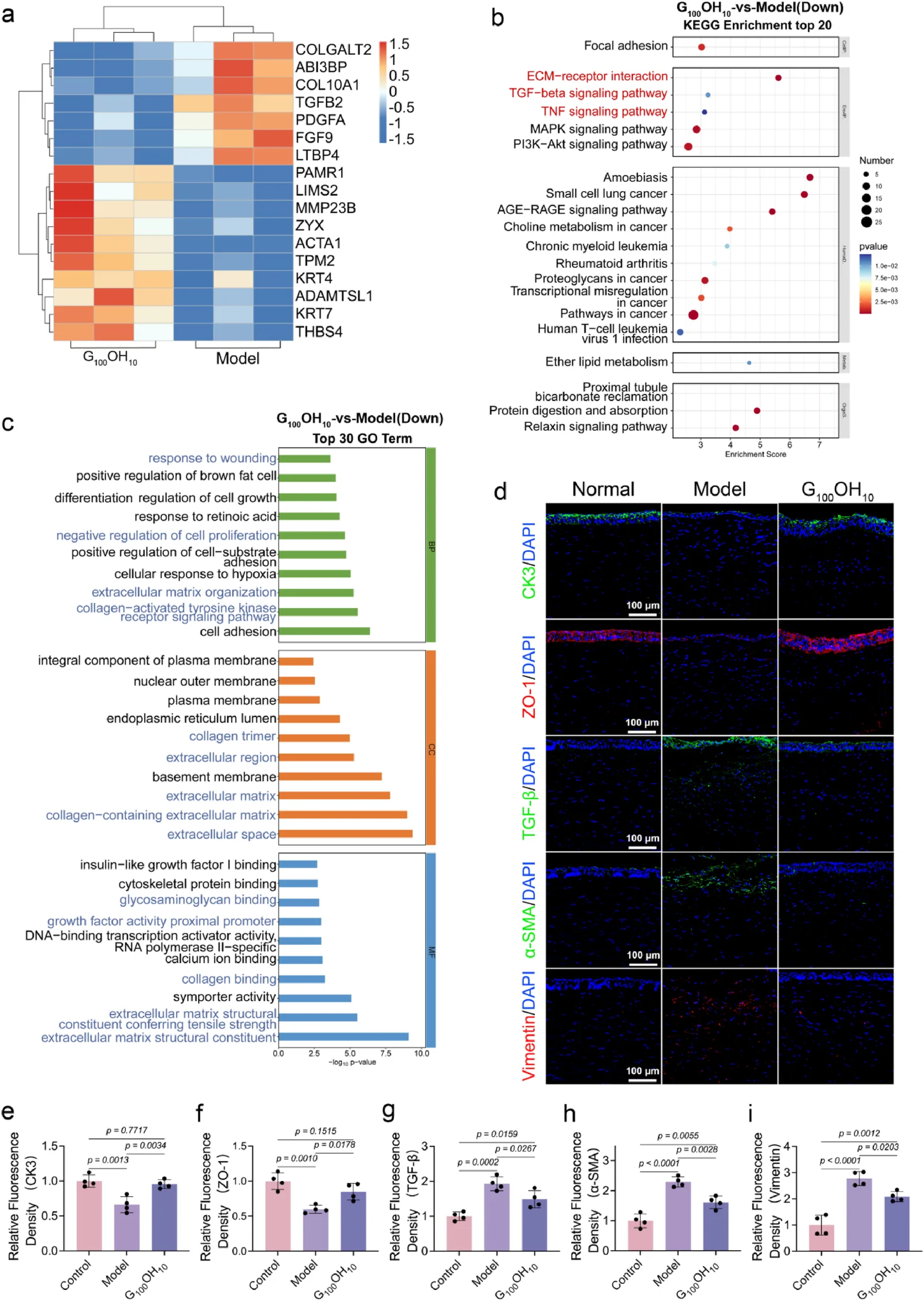
**Transcriptomic analysis of rabbit corneas from different groups.** (a) Heatmap-based cluster analysis. (b) Top 20 enriched KEGG pathways among downregulated DEGs. (c) Top 30 enriched GO terms among downregulated DEGs. (d) Immunofluorescence staining images of CK3, ZO-1, TGF-β, α-SMA, and vimentin expression in corneal tissues from different groups on postoperative day 30. Scale bar: 100 μm. Quantitative fluorescence analysis of (e) CK3, (f) ZO-1, (g) TGF-β, (h) α-SMA, and (i) vimentin expression in corneal tissues from different groups on postoperative day 30. Data are presented as mean ± SD (n = 4 independent samples) and analyzed by one-way ANOVA with multiple comparisons.

On postoperative day 30, immunofluorescence staining of CK3, ZO-1, TGF-β, α-SMA, and vimentin across the different groups was performed to assess the corneal epithelial and stromal remodeling. As shown in Fig. 4d–i, the normal corneal epithelium presented strong, uniform, and continuous CK3 expression, consistent with a mature stratified squamous architecture. In the Model group, the corneal epithelium exhibited sparse and discontinuous CK3 fluorescence, indicating immature epithelial regeneration. Notably, the corneal epithelium in the Hydrogel group showed markedly enhanced CK3 expression compared with the Model group, suggesting that G_100_OH_10_ hydrogel implantation promoted corneal epithelial maturation. Consistent with CK3 expression in corneal epithelium, ZO-1 was continuously distributed along epithelial cell junctions in normal corneal tissue, reflecting intact tight junction formation. ZO-1 staining was weak and discontinuous in the Model group, consistent with severe disruption of epithelial barrier integrity. By contrast, the Hydrogel group showed significantly enhanced ZO-1 fluorescence intensity and continuity, suggesting partial restoration of tight-junction integrity and epithelial barrier function. In addition, the Model group exhibited markedly increased TGF-β, α-SMA, and vimentin expression compared to normal corneal tissue, indicating stromal cell activation, mesenchymal phenotypic transition, and fibrotic remodeling. Notably, the expression of these fibrotic markers was substantially reduced in the Hydrogel group, approaching levels observed in normal corneal tissue. Together, these findings demonstrated that G_100_OH_10_ hydrogel implantation supported corneal remodeling by enhancing epithelial maturation and barrier restoration while suppressing injury-induced stromal fibrosis.

### 2.4 Rational design of HHC36-loaded hydrogel, *invitro* drug-release behavior and antibacterial efficacy

Deep corneal injury disrupts physiological barrier function and weakens ocular defense against microbial invasion, thereby markedly increasing the risk of infection [37]. Microbial infections, particularly bacterial infections, are generally associated with the corneal trauma. By secreting proteases and endo/exotoxins, bacteria can invade deeper corneal layers and activate excessive immune responses, leading to the release of pro-inflammatory mediators (*i.e.,* TNF-α, MMPs) and the recruitment of inflammatory cells to the injury site, thereby exacerbating corneal damage [38], [39], [40], [41]. To address this challenge, we further incorporated a cationic amphiphilic antimicrobial peptide (KRWWKWWRR; HHC36; Scheme S2, and Fig. S9) with broad-spectrum activity against drug-resistant Gram-positive and Gram-negative bacteria into the optimized hydrogel to afford a HHC36-loaded hydrogel [42].

The encapsulation efficiency of HHC36 in hydrogels with varying OHAMA concentrations was further evaluated. Pure GelMA exhibited an encapsulation efficiency of 77.11 ± 1.80%, comparable to that of G_100_OH_5_ (75.46 ± 1.93%), with no statistically significant difference. Increasing the OHAMA concentration further enhanced encapsulation efficiency, reaching 80.38 ± 2.22% and 86.07 ± 1.97% for G_100_OH_10_ and G_100_OH_20_, respectively. These findings suggest that OHAMA incorporation improves the HHC36 encapsulation capacity of GelMA-based hydrogels. This enhancement may be attributed to the formation of an interconnected copolymer network with a more favorable pore structure and increased availability of peptide-binding sites. Compared with pure GelMA, this composite network may facilitate peptide retention through physical confinement and additional polymer–peptide interactions, thereby improving encapsulation efficiency and potentially enhancing peptide retention stability. To visualize the spatial distribution of HHC36 within hydrogels of varying compositions, rhodamine B was used as a fluorescent surrogate for HHC36, and its distribution was assessed by three-dimensional fluorescence reconstruction (Fig. S10). All four hydrogel formulations (GelMA, G_100_OH_5_, G_100_OH_10_, and G_100_OH_20_) exhibited continuous and homogeneous fluorescence signals without noticeable aggregation, fluorescence-free regions, or localized enrichment. These findings indicate uniform probe distribution throughout the hydrogel networks, suggesting their potential to support spatially homogeneous antimicrobial peptide delivery in corneal repair applications.

We first evaluated the *in vitro* release behavior of HHC36 from various hydrogels in different release mediums. As shown in Fig. 5a, all hydrogels exhibited an initial burst release of HHC36 within the first 24 h in PBS. GelMA subsequently showed sustained release for up to 168 h, whereas G_100_OH_5_, G_100_OH_10_, and G_100_OH_20_ hydrogel prolonged the sustained-release period to 312, 418, and 720 h, respectively. GelMA displayed the fastest release profile, with a cumulative release of 51.7 ± 1.2% at 24 h. In contrast, G_100_OH_5_, G_100_OH_10_, and G_100_OH_20_ showed progressively slower release, with cumulative release values of 43.4 ± 0.7%, 36.9 ± 2.1%, and 30.3 ± 1.8%, respectively. The release profiles eventually reached plateaus, with final cumulative releases of 83.2 ± 3.9%, 81.0 ± 2.5%, 72.5 ± 2.3%, and 65.5 ± 1.7% for GelMA, G_100_OH_5_, G_100_OH_10_, and G_100_OH_20_ hydrogel, respectively. Notably, increasing OHAMA content in hydrogel progressively slowed HHC36 release. To better mimic the enzyme-rich *in vivo* microenvironment, *in vitro* HHC36 release from various hydrogels was further assessed in an enzyme-containing medium. As shown in Fig. 5b, all tested hydrogels exhibited significantly accelerated drug release, implying that enzymatic degradation efficiently disrupted the hydrogel network, reduced the diffusion barrier, and promoted HHC36 release. Within 24 h, GelMA and G_100_OH_5_ hydrogel showed a rapid drug release, achieving cumulative release values of 87.8 ± 1.9% and 82.2 ± 3.6%, respectively, whereas G_100_OH_10_ and G_100_OH_20_ hydrogel exhibited slower release, reaching only 64.2 ± 3.2% and 42.6 ± 3.9% cumulative release, respectively. Based on these results, HHC36-loaded G_100_OH_10_ hydrogel (HHC36@Gel) was selected for subsequent investigations. The incorporation of HHC36 resulted in a slight decrease in the light transmittance of HHC36@Gel compared with G_100_OH_10_ hydrogel (Fig. 5c). The refractive index of HHC36@Gel was 1.351 ± 0.0015, slightly higher than that of G_100_OH_10_ but still comparable to that of the native cornea (Fig. S11a). After reaching swelling equilibrium at 12 h, HHC36@Gel exhibited a swelling ratio of 18.59 ± 0.94%, which was similar to that of G_100_OH_10_ (Fig. S11b). The incorporation of HHC36 did not significantly affect the compressive properties of G_100_OH_10_, with a compressive stress of 0.55 MPa (Fig. S11c). Likewise, the elastic modulus of HHC36@Gel was 74.62 ± 8.09 KPa, showing no significant difference from that of G_100_OH_10_ (Fig. S11d). Additionally, HHC36@Gel exhibited a gelation time of 7 s, and its storage modulus reached 5,870 Pa at 60 s, slightly lower than that of G_100_OH_10_ hydrogel (Fig. 5d). Overall, the incorporation of HHC36 had only a minor effect on the optical and mechanical properties of the hydrogel, likely because of its low incorporation level (<1.79 wt%), which resulted in the formation of only a limited number of Schiff-base bonds between the lysine residues in HHC36 and OHAMA. Moreover, the efficient release of HHC36 from the hydrogel further reflected the dynamic nature of the Schiff-base bonds, which are inherently capable of reversible cleavage and reformation. Importantly, this dynamic behavior may enable the hydrogel network to adapt more effectively to the continuously evolving microenvironment during *in vivo* tissue remodeling.

**Fig. 5:**
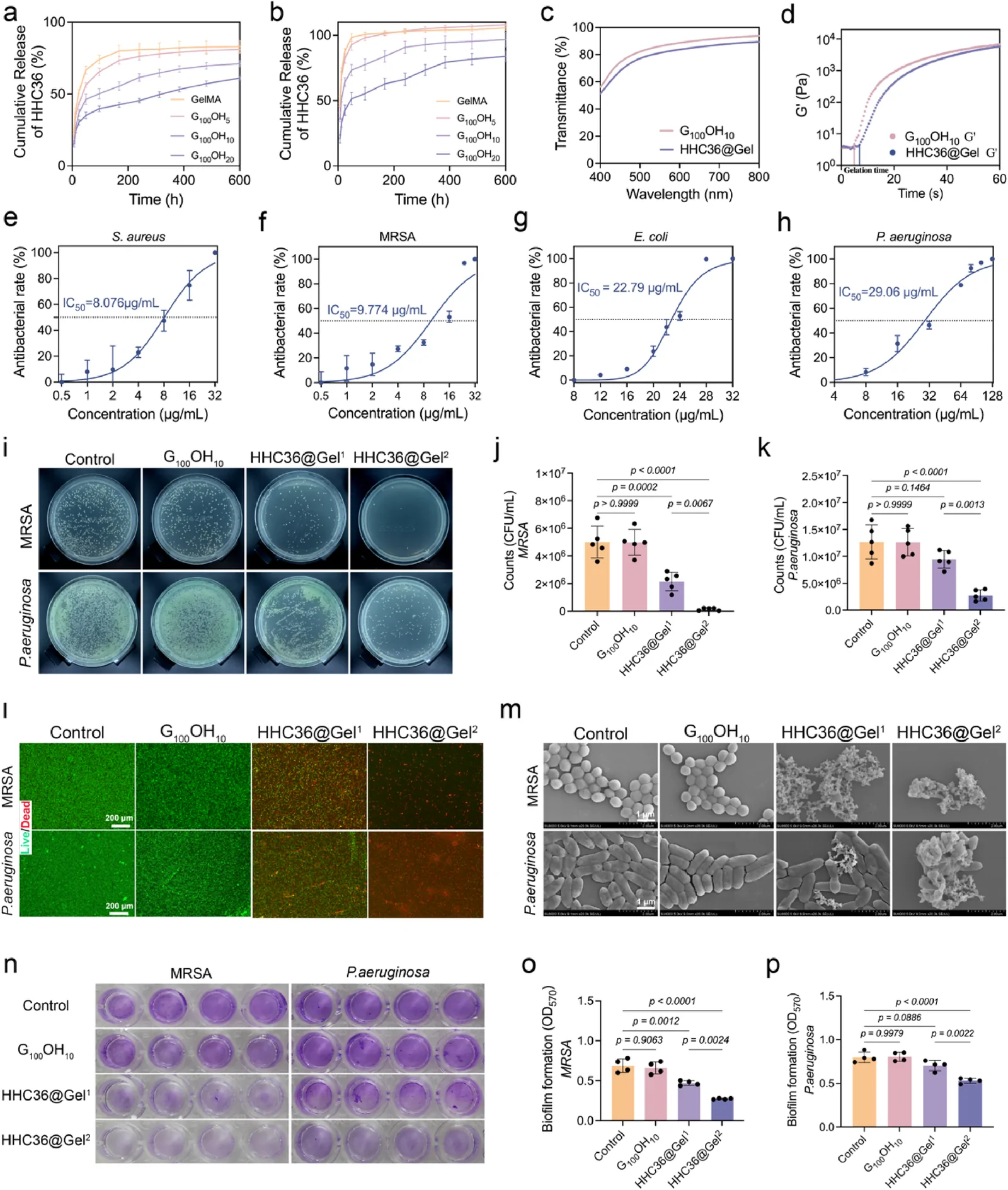
***In vitro* drug release and *in vitro* antimicrobial activity of HHC36-loaded hydrogel.***In vitro* release profiles of HHC36 from the different hydrogels in (a) PBS solution and (b) PBS containing with 5 U/mL hyaluronidase and ≥ 5 CDU/mL collagenase. (c) Transmittance of G_100_OH_10_ hydrogel and HHC36-loaded G_100_OH_10_ hydrogel (HHC36@Gel^2^). (d) Time-dependent storage modulus of G_100_OH_10_ hydrogel and HHC36-loaded G_100_OH_10_ hydrogel (HHC36@Gel^2^). Half-maximal inhibitory concentration (IC_50_) of HHC36 against (e) *S. aureus*, (f) MRSA, (g) *E. coli*, and (h) *P. aeruginosa*. (i) Representative photographs of bacterial colonies on LB agar plates after different treatments for 24 h. Quantification of colony-forming units (CFUs) of (j) MRSA and (k) *P. aeruginosa*. (l) Live/dead staining images of MRSA and *P. aeruginosa* after different treatments for 24 h. Live bacteria are shown in green, and dead bacteria are shown in red. Scale bar: 200 μm. (m) SEM images of bacterial strains after different treatments for 24 h. Scale bar: 1 μm. (n) Crystal violet staining of bacterial biofilms from different groups at 48 h. Quantification of OD_570_ values of (o) MRSA and (p) *P. aeruginosa* biofilms at 48 h. HHC36@Gel^1^ (1 mg/mL HHC36); HHC36@Gel^2^ (2 mg/mL HHC36); Data are presented as mean ± SD [(a, b, e–h, o, p) n = 4 independent samples; (j, k) n = 5 independent samples] and analyzed by one-way ANOVA with multiple comparisons.

The antimicrobial activity of HHC36 was first evaluated by determining its IC_50_ using the broth microdilution method. As shown in Fig. 5e–h, HHC36 exhibited potent antibacterial activity against both Gram-positive *S. aureus* and Gram-negative *E. coli*, with IC_50_ values of 8.076 and 22.790 μg/mL, respectively. Notably, HHC36 retained strong activity against clinically challenging strains, including MRSA and *P. aeruginosa*, with IC_50_ values of 9.774 and 29.060 μg/mL, respectively. To further investigate the stability of HHC36 under ocular physiological conditions, the peptide was incubated at 37 °C in PBS, lysozyme-free simulated tear fluid (STF), or STF containing 0.01% lysozyme. Samples were collected after 2 h, 24 h, 7 d, 14 d and 30 d, and residual antibacterial activity was assessed by determining IC_50_ values against MRSA and *P. aeruginosa* (Fig. S12). During the initial 24 h, no significant changes in IC_50_ values were observed in any medium, indicating that HHC36 retained its antibacterial activity under all tested conditions. However, prolonged incubation for 14 and 30 d resulted in varying degrees of activity loss, with a more pronounced reduction in efficacy against *P. aeruginosa* than against MRSA. Notably, HHC36 exhibited greater activity loss in lysozyme-containing STF than in PBS or lysozyme-free STF, suggesting that the presence of lysozyme may contribute to reduced peptide stability during prolonged incubation. Overall, these results demonstrate that HHC36 maintains its antibacterial activity during short-term exposure to simulated ocular conditions, although prolonged incubation, particularly in the presence of lysozyme, is associated with a gradual decline in antibacterial efficacy. To assess the long-term storage stability of HHC36, the structural integrity and antibacterial activity of the peptide were evaluated after three months of low-temperature storage in a lyophilized state using LC–MS and IC_50_ assays, respectively (Fig. S13). LC–MS analysis confirmed that the molecular weight of stored HHC36 matched its theoretical value, with no detectable impurity, degradation, or aggregation peaks and a purity exceeding 95%. These results indicate that HHC36 maintained its structural integrity during storage. Consistently, the IC_50_ values of stored HHC36 against MRSA and *P. aeruginosa* did not differ significantly from those of freshly prepared HHC36, demonstrating preserved antibacterial activity. Together, these findings suggest that low-temperature lyophilized storage effectively maintains the structural integrity and antibacterial efficacy of HHC36 for at least three months.

Following by co-incubation with MRSA or *P. aeruginosa* for 24 h, both low- and high-concentration HHC36@Gel formulations (HHC36@Gel^1^ (1 mg/mL HHC36) and HHC36@Gel^2^ (2 mg/mL HHC36)) exhibited substantial antibacterial efficacy, as evidenced by the significant reduction in colony formation on agar plates (Fig. 5i). Quantitative analysis further demonstrated that the antibacterial effect increased with HHC36 concentration, although the inhibition against *P. aeruginosa* was slightly weaker than that against MRSA (Fig. 5j and k). Consistently, OD_600_ measurements showed rapid bacterial growth in the Control and Hydrogel groups, whereas the HHC36@Gel groups displayed significantly lower OD values in a concentration-dependent manner (Fig. S14). Furthermore, Live/dead staining assay revealed that most bacteria from Control and Hydrogel groups remained viable, as evidenced by the strong green fluorescence signal (Fig. 5l). In contrast, bacterial viability progressively decreased in the HHC36@Gel^1^ and HHC36@Gel^2^ groups, as indicated by the increasingly prominent red fluorescence signal corresponding to dead bacteria. SEM observation (Fig. 5m) indicated that MRSA in the Control and Hydrogel groups retained its characteristic grape-like clustered morphology with smooth, intact surfaces, whereas *P. aeruginosa* displayed a rod-shaped morphology with preserved outer membrane integrity. Notably, bacteria in the HHC36@Gel^1^ group exhibited rough and damaged membranes, together with cell collapse, shrinkage, deformation and adhesion. These morphological disruptions were more pronounced in the HHC36@Gel^2^ group, where bacterial rupture, lysis, cytoplasmic leakage and dense extracellular exudates were observed. Since biofilm formation is closely associated with persistent bacterial infection and antibiotic resistance [43], the antibiofilm activity of various formulations was evaluated by crystal violet staining. After 48 h of co-culture with MRSA or *P. aeruginosa*, the Control and Hydrogel groups formed dense, dark-purple biofilms covering the culture plates (Fig. 5n). Conversely, both HHC36@Gel^1^ and HHC36@Gel^2^ group significantly suppressed biofilm formation and reduced staining intensity, with the strongest inhibitory effect observed in the HHC36@Gel^2^ group. Quantitative OD_570_ analysis further identified that the HHC36@Gel^2^ group exhibited the strongest anti-biofilm capacity among all tested formulations (Fig. 5o and p). Taken together, these results demonstrate that HHC36@Gel possesses potent antibacterial efficacy and holds considerable promise as a therapeutic scaffold for the management of corneal injuries complicated by bacterial infection.

To evaluate the cytocompatibility of HHC36, cell viability was assessed using the MTT assay following exposure to different HHC36 concentrations (6.25–200 μg/mL) for 1, 3, and 5 days (Fig. S15). No significant reduction in cell viability was observed at any tested concentration or time point compared with the untreated control, indicating favorable cytocompatibility within the investigated concentration range. The selective antibacterial activity of HHC36 may be attributed to differences in membrane composition and surface charge between bacterial and mammalian cells. Negatively charged bacterial membranes favor electrostatic interactions with cationic antimicrobial peptides, whereas the predominantly zwitterionic outer leaflet of mammalian cell membranes exhibits weaker electrostatic affinity, potentially limiting peptide-induced cytotoxicity. These findings suggest that HHC36 exhibits favorable cellular tolerance within the tested concentration range. In addition to peptide cytocompatibility, the biological safety of hydrogel degradation products is essential for ocular applications. GelMA, a methacryloyl-modified gelatin derivative, is expected to undergo degradation into peptide fragments and amino acids that can be metabolized through physiological pathways. Similarly, OHAMA is derived from oxidized and methacryloyl-modified hyaluronic acid, a naturally occurring component of the extracellular matrix. Its degradation may generate hyaluronic acid-derived oligosaccharides, some of which have been associated with the regulation of cell migration and tissue repair. Collectively, these findings support the cytocompatibility of HHC36 and the potential biocompatibility of the GelMA/OHAMA matrix, highlighting the promise of this composite hydrogel system for ocular surface applications.

### 2.5 HHC36@Gel^2^ facilitating corneal stromal remodeling with reduced scar formation on deep corneal defect complicated by *P. aeruginosa* infection

*P. aeruginosa* is a Gram-negative bacillus with a strong capacity for biofilm formation, enabling it to establish a protective microenvironment within the corneal stroma. In addition, *P. aeruginosa* secretes various proteases that accelerate collagen degradation and tissue liquefaction. Given the potent *in vitro* antibacterial efficacy of HHC36@Gel^2^ formulation, we thereafter evaluated its therapeutic potential *in vivo* using a rabbit model of deep corneal defect complicated by *P. aeruginosa* infection (Fig. 6a). On postoperative day 1, all groups exhibited varying degrees of inflammation, including conjunctival congestion and edema, corneal edema, and iris congestion, with the Model group showing the most severe inflammatory response (Fig. 6b). By day 7, inflammation had further progressed in the Model group, accompanied by exacerbated corneal edema and pronounced bulbar conjunctival and iris congestion. In contrast, clinical signs of corneal edema and iris congestion were markedly alleviated in both Gel + LVX and HHC36@Gel^2^ groups, with only mild iris congestion observed in HHC36@Gel^2^ group. Despite incomplete re-epithelialization in all groups at day 7, corneal epithelial repair was substantially enhanced in the Gel + LVX and HHC36@Gel^2^ groups compared with the Model group. By day 30, the Model group remained corneally hazy, with extensive neovascularization extending into the defect area, whereas corneas appeared clear and transparent with no apparent neovascularization in both Gel + LVX and HHC36@Gel^2^ groups. Similarly, AS-OCT imaging revealed progressive worsening of corneal edema in the Model group, accompanied by an increase in corneal thickness from day 1 to day 7 (Fig. 6c). By day 30, mass-like hyperreflective structures consistent with neovascularization were observed in the central cornea. Notably, corneas in the Gel + LVX and HHC36@Gel^2^ groups showed only mild early edema and thickening, which had largely resolved by day 30, with near-normal morphology and thin, uniform hyperreflective scar signals remaining in the defect area. Bacterial colony assays performed on day 7 demonstrated that both Gel + LVX and HHC36@Gel^2^ groups significantly reduced bacterial burdens compared with the Model group (Fig. 6d and f). This finding strongly suggested that HHC36@Gel^2^ not only filled the corneal defect and prevented infection but also achieved antibacterial efficacy comparable to that of the clinically used antibiotic regimen (Gel + LVX).

**Fig. 6:**
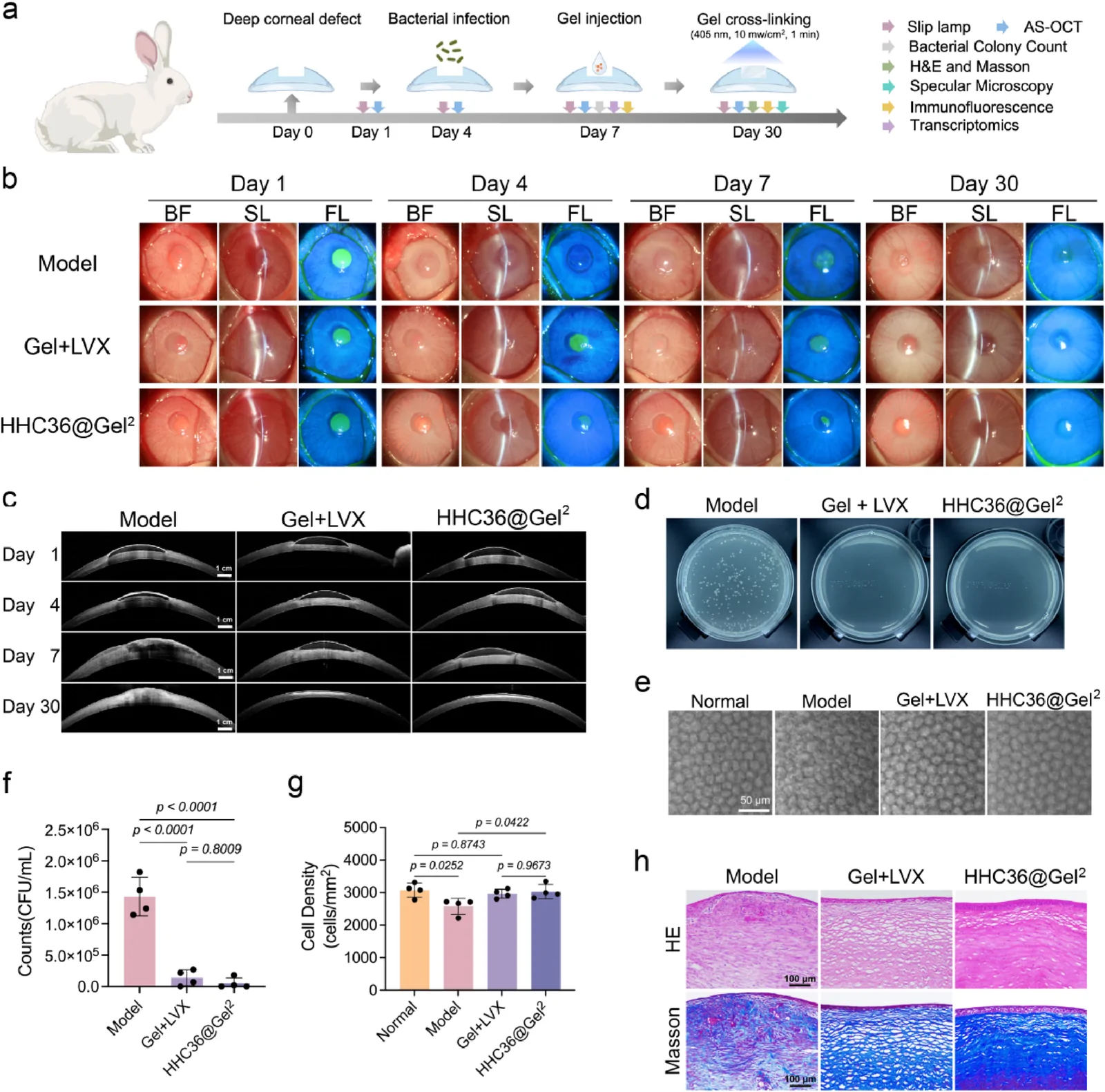
***In vivo* therapeutic efficacy of HHC36@Gel^2^ in a rabbit model of deep corneal stromal defects complicated by *P. aeruginosa* infection.** (a) Schematic illustration of model establishment, treatment protocol, and outcome assessment. (b) Representative bright-field (BF), slit-lamp (SL), and fluorescein staining (FL) images of rabbit eyes from different groups. The Model group received no treatment after inoculation of the corneal defect with *P. aeruginosa*. The Gel + LVX group received G_100_OH_10_ hydrogel treatment combined with levofloxacin eye drops (50 μL, 4.88 mg/mL) three times daily after *P. aeruginosa* inoculation. The HHC36@Gel^2^ group received HHC36-loaded hydrogel (2 mg/mL HHC36) treatment after *P. aeruginosa* inoculation. (c) AS-OCT images of corneal stromal defects complicated by *P. aeruginosa* infection from different groups. (d) Representative photographs of bacterial colonies isolated from different corneas on postoperative day 7. (e) Representative corneal specular microscopy images of the corneal endothelium from different groups on postoperative day 30. (f) Quantification of bacterial colony counts from different groups on postoperative day 7. (g) Quantification of corneal endothelial cell density from different groups on postoperative day 30. (h) H&E and Masson staining images of corneal tissues from different groups. Scale bar: 100 μm. Data are presented as mean ± SD (n = 4 independent samples) and analyzed by one-way ANOVA with multiple comparisons.

On postoperative day 30, microscopic analysis of corneal endothelial cells (Fig. 6e and g) showed pronounced morphological abnormalities and a significant reduction in endothelial cell density in the Model group relative to the Normal group, suggesting substantial endothelial injury resulting from persistent infection and inflammation. Notably, both Gel + LVX and HHC36@Gel^2^ groups exhibited endothelial cell density and morphology comparable to those of the Normal group, implying that Gel + LVX and HHC36@Gel^2^ treatment effectively suppressed ocular infection and inflammation while preserving the corneal endothelium. H&E staining (Fig. 6h) showed that the Model group exhibited the destroyed corneal architecture and abundant disorganized collagen fibers within the defect area, accompanied by prominent neovascular clusters and extensive inflammatory cell infiltration. By contrast, both Gel + LVX and HHC36@Gel^2^ groups exhibited a near-normal corneal architecture, with minimal neovascularization and negligible inflammatory cell infiltration. Masson staining (Fig. 6h) further demonstrated enhanced corneal stromal remodeling in the HHC36@Gel^2^ group relative to the Model and Gel + LVX groups, characterized by a regenerated stratified squamous epithelium and well-organized, densely packed collagen fibers, resembling the native corneal lamellar structure. Although *P. aeruginosa* is associated with a higher risk of corneal perforation than many other common ocular pathogens in clinical settings, the use of this organism alone to establish the infection model is insufficient to comprehensively evaluate the therapeutic efficacy of the hydrogel against Gram-positive bacterial infections. In future studies, we will further assess the therapeutic efficacy of the hydrogel using mixed-infection models.

To further analyze the potential molecular mechanism of HHC36@Gel^2^ on the promotion of corneal stromal remodeling under the condition of bacterial infection, transcriptomic analysis was performed on postoperative day 7. As shown in Fig. 7a, cluster heatmap analysis revealed distinct expression profiles of genes associated with inflammation, immunity, and extracellular matrix remodeling between the Model and HHC36@Gel^2^ groups. In the Model group, the expression of several pro-inflammatory and chemotactic genes, including *TNF*, *IL1B*, *CCL2*, *CCL7*, *GRO-A*, and *GRO-B*, was elevated, accompanied by upregulation of genes associated with leukocyte recruitment and adhesion, such as *ICAM1*, *ITGB2*, and *ITGAD*. Additionally, genes involved in fibrosis and pathological extracellular matrix remodeling, including *TGFB2*, *IL11*, *MMP1*, and *MMP3*, exhibited increased expression. In contrast, HHC36@Gel^2^ treatment was associated with upregulated expression of *IL1R2*, *SERPINB1*, *SLURP1*, and *COL4A6*, which are implicated in inflammatory regulation, tissue protection, and extracellular matrix homeostasis. Collectively, these transcriptional changes suggest that HHC36@Gel^2^ modulates the inflammatory microenvironment of infected corneas, potentially mitigating persistent inflammation and pathological fibrotic remodeling. Consistently, KEGG enrichment analysis of genes downregulated in the HHC36@Gel^2^ group revealed significant enrichment in infection-, inflammation-, and immune-related pathways, including cytokine–cytokine receptor interaction, chemokine signaling, and JAK–STAT signaling pathways (Fig. 7b). GO enrichment analysis (Fig. 7c) further supported these findings and the enriched biological process terms were mainly associated with immune response, inflammatory response, neutrophil chemotaxis, leukocyte migration, and extracellular matrix organization. Altogether, these transcriptomic results demonstrated that the HHC36@Gel^2^ treatment might be able to downregulate genes associated with inflammatory activation and immune cell recruitment in infected corneas, thereby limiting infection-induced amplification of the inflammatory cascade and promoting the corneal stromal remodeling.

**Fig. 7:**
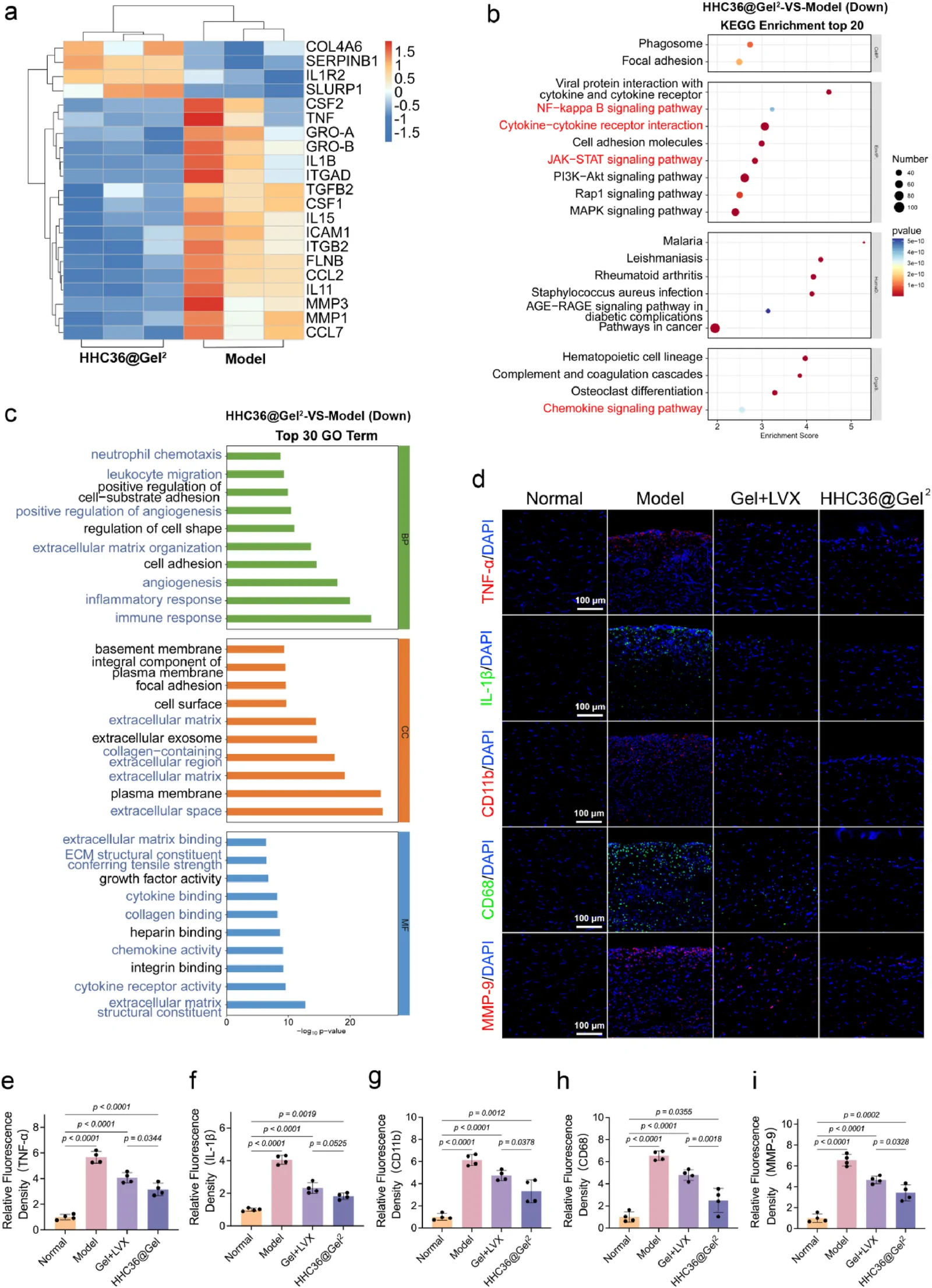
**Transcriptomic analysis of rabbit corneas with stromal defects complicated by *P. aeruginosa* infection.** (a) Heatmap-based clustering analysis of DEGs. (b) Top 20 enriched KEGG pathways among downregulated DEGs. (c) Top 30 enriched GO terms among downregulated DEGs. (d) Representative immunofluorescence staining images of TNF-α, IL-1β, CD68, CD11b, and MMP-9 expression in corneal tissues from different groups on postoperative day 7. Scale bar: 100 μm. Quantitative fluorescence analysis of (e) TNF-α, (f) IL-1β, (g) CD11b, (h) CD68, and (i) MMP-9 expression in corneal tissues from different groups on postoperative day 7. Data are presented as mean ± SD (n = 4 independent samples) and analyzed by one-way ANOVA with multiple comparisons.

Immunofluorescence staining assays were thereafter performed to assess pro-inflammatory cytokines expression and inflammatory cell infiltration in corneal tissues. As shown in Fig. 7d–i, the Model group showed markedly increased expression of the pro-inflammatory cytokines IL-1β and TNF-α, and enzyme MMP-9, together with enhanced infiltration of CD11b^+^ and CD68^+^ inflammatory cells, compared with the Normal group, indicating the excessive inflammatory response after injury. Both Gel + LVX and HHC36@Gel^2^ treatments significantly suppressed the expression of the pro-inflammatory cytokines IL-1β and TNF-α, as well as MMP-9, and concomitantly reduced the infiltration of CD11b^+^ and CD68^+^ inflammatory cells. Notably, HHC36@Gel^2^ group exerted a stronger inhibitory effect than Gel + LVX group, further reducing TNF-α and MMP-9 levels as well as the infiltration of CD11b^+^ and CD68^+^ inflammatory cells. These findings suggested that HHC36@Gel^2^ more effectively suppresses excessive inflammation, thereby creating a microenvironment more conducive to corneal stromal remodeling. Given that excessive inflammation can drive the differentiation of corneal keratocytes into myofibroblasts, thereby disrupting normal ECM secretion and organization and promoting scar formation [44], [45], [46], [47], we next assessed α-SMA and vimentin expression to evaluate stromal fibrosis (Fig. S16). As expected, the Model group exhibited the highest expression of α-SMA and vimentin among all groups, confirming that bacterial-infected injury triggered abnormal fibrotic remodeling in the corneal stroma. Both Gel + LVX and HHC36@Gel^2^ group significantly reduced α-SMA and vimentin expression compared with the Model group, with HHC36@Gel^2^ group producing the most pronounced suppression of both fibrotic markers. Importantly, HHC36 is immobilized within the dual-crosslinked hydrogel network, allowing prolonged retention at the stromal defect and sustained *in situ* antibacterial activity. In contrast, small-molecule levofloxacin readily diffuses from the lesion, potentially shortening its local therapeutic duration and allowing residual pathogens to sustain inflammation, stromal damage, and scar formation. Overall, the clinical, transcriptomic and histopathological results demonstrate that HHC36@Gel^2^ provides potent antibacterial efficacy and mitigates excessive inflammation by suppressing pro-inflammatory cytokine expression and inflammatory cell infiltration, thereby facilitating corneal stromal remodeling with reduced scar formation. Dynamic Schiff-base bonds between HHC36 and OHAMA enable sustained peptide release without pronounced burst release, while low-dose HHC36 loading preserves the crosslinking density and structural stability of the GelMA/OHAMA network. Released HHC36 exerts antibacterial effects and mitigates infection-induced inflammation, whereas the stable hydrogel microenvironment regulates corneal stromal cell proliferation, suppresses fibrosis, and promotes organized collagen remodeling. Together, these synergistic effects facilitate antibacterial and anti-inflammatory activity and scarless corneal remodeling, as supported by transcriptomic and immunofluorescence analyses.

### 2.6 *In vivo* biosafety of G_100_OH_10_ hydrogel

To assess the long-term *in vivo* biosafety of G_100_OH_10_ hydrogel implantation, the rabbit from normal and Hydrogel (G_100_OH_10_ hydrogel) group were sacrificed and major organs including heart, liver, spleen, lung and kidney were harvested on day 90 post-implantation for H&E staining. As shown in Fig. S17, major organs, including the heart, liver, spleen, lung, and kidney, from both the Normal and Hydrogel groups exhibited intact tissue architecture and well-preserved cellular morphology, with no evident degeneration, necrosis, or inflammatory cell infiltration. These findings indicate that prolonged *in vivo* retention of the G_100_OH_10_ hydrogel does not cause detectable toxicity and demonstrates favorable biosafety.

## 3 Discussion

Developing *in situ* hydrogel-based substitutes that can replace corneal transplantation and be applied to severe corneal trauma has become an urgent clinical need in ophthalmology [48]. As ideal corneal substitutes, hydrogel-based materials should integrate several key properties, including optical transparency, tunable biomechanical performance, and cytocompatibility, to recapitulate native corneal physiology. To mimic the compositions of corneal stroma, we selected hyaluronic acid (HA) and gelatin derivatives as substrates to engineer an optically transparent and biomechanically tissue-matched dual-crosslinkable hydrogel capable of enabling cell-free corneal stromal remodeling. Although HA- and gelatin-based hydrogels can recapitulate key optical and biochemical features of the corneal ECM, making them attractive candidates for corneal stromal remodeling, these hydrogels are generally limited by rapid degradation and insufficient mechanical strength. To overcome these limitations, chemical functionalization of HA and gelatin with reactive groups enables crosslinking and permits precise tuning of hydrogel properties to match those of the native cornea. Herein, HA was chemically oxidized and methacrylated to generate oxidized hyaluronic acid methacryloyl (OHAMA). By combining OHAMA with gelatin methacryloyl (GelMA) initiated at 405 nm visible-light irradiation in the presence of the photoinitiator LAP, we developed a series of hydrogels with tunable optical transparency, biomechanical strength and stable wet adhesion. After optimization, the GelMA/OHAMA hydrogels rapidly formed *in situ* and exhibited visible-light transmittance exceeding 80%, comparable to that of the native cornea. The refractive index of hydrogels was very close to that of the native cornea (approximately 1.376), which is critical for visual restoration. Because biomechanical compatibility is essential for scarless corneal remodeling, hydrogels designed for corneal stromal repair should exhibit mechanical properties that match those of native corneal tissue. By varying the crosslinking strategy, robust GelMA/OHAMA hydrogels with moduli ranging from 12.30 ± 2.00 kPa to 134.40 ± 30.47 kPa were obtained. Notably, shifting from single photo-crosslinking to a dual-crosslinking strategy enabled fine-tuned regulation of the balance between rigidity and flexibility. Accordingly, the swelling ratio of the hydrogels could be finely tuned between 29.73 ± 3.56% and 10.17 ± 3.99% by modulating the OHAMA content. Excessive swelling of hydrogel-based corneal substitutes may induce edema, impair wound healing and distort corneal curvature. Therefore, precise control over swelling is essential for the design of reliable and therapeutically effective hydrogel-based corneal substitutes.

Given that the corneal surface is covered by a tear film, hydrogel-based corneal substitutes must achieve stable adhesion in a highly hydrated environment to ensure integration with surrounding tissue and prevent displacement or detachment. The proposed GelMA/OHAMA hydrogels formed robust adhesive interfaces in the wet corneal microenvironment through dual covalent crosslinking mediated by Schiff-base reactions and photopolymerization. In a rabbit model of deep corneal stromal defects, the optimized G_100_OH_10_ hydrogel maintained stable adhesion for 30 days, thereby meeting the requirements for corneal stromal remodeling. Similar to other natural hydrogels, such as chitosan and collagen, G_100_OH_10_ hydrogel exhibited negligible cytotoxicity toward L929 and HCEC cells, while effectively promoting HCEC proliferation and migration. After *in situ* gelation at the injured site, G_100_OH_10_ hydrogel filled the stromal defect and exhibited high transparency, a low swelling ratio, favorable biomechanical properties, and robust adhesive performance, thereby markedly promoting corneal re-epithelialization and stromal remodeling while reducing inflammation and scar formation. Under physiological conditions, corneal stromal transparency is maintained by highly organized collagen fibrils; however, severe corneal trauma induces keratocyte-to-myofibroblast transformation, leading to deposition of disorganized collagen fibers and extracellular matrix components and ultimately the formation of opaque scar tissue. Following G_100_OH_10_ hydrogel implantation, the hydrogel recapitulated key functions of the stromal ECM, promoting re-epithelialization and restoration of the epithelial barrier, as evidenced by enhanced CK3 and ZO-1 expression. Moreover, it enabled scarless stromal remodeling by reducing TGF-β, α-SMA and vimentin expression and promoting the formation of collagen fibrils with uniform diameter and regular interfibrillar spacing. Consistent with these findings, Huang and colleagues reported a bioactive hydrogel based on gelatin and anionic heparin for scarless corneal repair. This hydrogel restricted the influx of inflammatory and fibrotic cytokines, such as IL-1, TGF-β, and PDGF-BB, into the stroma, thereby suppressing keratocyte apoptosis and myofibroblast differentiation [27].

To address the dual challenges of corneal scarring and bacterial infection, we further incorporated a cationic antimicrobial peptide (HHC36) into G_100_OH_10_ hydrogel to generate HHC36@Gel. Notably, the physical incorporation of HHC36 had minimal effects on the optical and biomechanical properties of hydrogel. The resulting HHC36@Gel enabled sustainable peptide release, thereby maintaining therapeutic concentrations and reducing dosing frequency. Integration of HHC36 into the G_100_OH_10_ hydrogel endowed HHC36@Gel with robust antibacterial activity, as demonstrated by reduced bacterial colony formation *in vitro* and suppressed biofilm formation. By integrating of HHC36 into G_100_OH_10_ hydrogel imparts the HHC36@Gel robust antibacterial ability by the reduction of colony formation *in vitro* and the suppression of bio-film formation. Using a rabbit model of deep corneal defects complicated by *P. aeruginosa* infection, we demonstrated that HHC36@Gel^2^ treatment not only effectively eradicated bacteria but also modulated the immune microenvironment by suppressing IL-1β, TNF-α and MMP-9 expression and reducing the infiltration of CD11b^+^ and CD68^+^ inflammatory cells, ultimately enabling scarless stromal remodeling. In addition to confirming the involvement of the TGF-β/α-SMA-mediated fibrotic pathway, a well-established and conserved mechanism in corneal repair, our findings suggest that HHC36 can indirectly suppress inflammation-driven fibrotic overactivation through its antibacterial activity, thereby attenuating TGF-β/α-SMA/vimentin signaling and promoting more organized collagen remodeling in infected corneal stromal defects. Remarkably, HHC36@Gel^2^ exhibited superior *in vivo* performance compared with clinically used antibiotic treatment (Gel + LVX). Similarly, Han and co-workers developed a Janus hydrogel with an asymmetric architecture by integrating heparin and poly-lysine, thereby achieving synergistic antibacterial and pro-regenerative bioactivities for comprehensive corneal injury management [24]. Compared with previously reported biomaterials for corneal repair, the dual-crosslinked hydrogel developed in this study offers advantages in mechanical adaptability, antibacterial activity, and extracellular matrix remodeling. Although GelMA-based hydrogels exhibit good cytocompatibility, their weak mechanical properties, excessive swelling, and rapid degradation may limit long-term support for deep corneal stromal defects. These limitations may lead to disorganized collagen deposition and scarring. Moreover, conventional corneal scaffolds often lack intrinsic antibacterial activity, while antibiotic-loaded hydrogels may cause burst release, cytotoxicity, and antibiotic resistance.

Although dual-crosslinking strategies have been widely applied in hydrogel systems, the novelty of this study lies in integrating a GelMA/OHAMA dual-crosslinking network with the antimicrobial peptide HHC36 to simultaneously address infection control, tissue remodeling, and fibrosis inhibition in infected deep corneal stromal defects. By optimizing the GelMA-to-OHAMA ratio, the mechanical and optical properties of the hydrogel were precisely tuned to create a cornea-mimetic network with both structural stability and dynamic degradability. Moreover, the incorporation of HHC36 into this dual-crosslinking system endows the hydrogel with antimicrobial functionality, providing a promising therapeutic strategy for the repair of infected deep corneal stromal defects.

Despite the outstanding performance of the proposed hydrogel in both uninfected and infected models of deep corneal stromal defects, its clinical translation remains challenging. First, hydrogel standardization and sterilization represent substantial technical hurdles because of batch-to-batch variability and the risk of material degradation. Second, the ocular surface microenvironment is highly complex and dynamic in clinical practice, which may lead to a mismatch between hydrogel properties, including drug-release behavior and structural stability, and the temporal progression of corneal repair. Finally, engineering hydrogels that facilitate precise cellular alignment and recapitulate the hierarchical lamellar architecture of the native cornea remains a major challenge for achieving tissue integration and functional restoration. To overcome these formidable bottlenecks, several strategic countermeasures may be considered. Real-time rheological monitoring could serve as a robust batch-release quality-control criterion, helping to ensure consistent gelation performance across production lots. With respect to sterilization, filtration of the precursor solutions represents a mild yet effective approach. In parallel, lyophilization of the aldehyde-functionalized polymer (OHAMA) into a solid dosage form, followed by ex tempore reconstitution immediately prior to administration, may substantially extend shelf life while preserving its chemical reactivity. In addition, rheological properties relevant to clinical handling, including the injectability window and shear-thinning behavior during needle passage, should be systematically characterized to ensure that the gelation kinetics are compatible with routine clinical injection workflow. For example, a setting time of less than 60 s at physiological temperature may provide a practical balance between ease of administration and rapid *in situ* gel formation.

## 4 Conclusion

In conclusion, we rationally designed and developed an optically and biomechanically corneal tissue-matched dual-crosslinkable *in situ* hydrogel based on GelMA and OHAMA for cell-free corneal stromal remodeling. By the integration of chemical crosslinking and photo-crosslinking strategies, the optical and biomechanical properties of hydrogels were precisely tuned. The optimized G_100_OH_10_ hydrogel exhibited high transparency, a refractive index of approximately 1.35, and an elastic modulus of 83.8 ± 13.4 kPa, closely mimicking those of native cornea. After application to a rabbit model of deep corneal stromal defects, the G_100_OH_10_ hydrogel closely conformed to the defect geometry, established stable adhesion, and persisted at the injury site for an extended period. By recapitulating ECM functions, the G_100_OH_10_ hydrogel promoted corneal scarless stromal remodeling, as evidenced by restoration of the epithelial barrier through enhanced CK3 and ZO-1 expression, together with orderly collagen remodeling within the stromal layer by reduced TGF-β, α-SMA, and vimentin expression. Incorporation of the antibacterial peptide HHC36 into the hydrogel yielded HHC36@Gel, which exhibited robust antibacterial activity without compromising its intrinsic regenerative capacity. In a rabbit model of deep corneal stromal defects complicated by *P. aeruginosa* infection, HHC36@Gel not only eradicated bacterial infection but also modulated the inflammatory microenvironment by suppressing IL-1β, TNF-α and MMP-9 expression and reducing CD11b^+^ and CD68^+^ inflammatory-cell infiltration, thereby enabling scarless corneal stromal remodeling. Overall, this optically and biomechanically corneal tissue-matched dual-crosslinkable *in situ* hydrogel represents a promising strategy for corneal stromal remodeling and offers new hope for the treatment of infectious corneal trauma.

## 5 Experimental section

### 5.1 Materials

Hyaluronic acid (HA, 200-400 kDa, H293507, Aladdin®), methacrylate anhydride, sodium periodate, and lithium phenyl-2,4,6-trimethylbenzoylphosphinate (LAP) were purchased from Aladdin® (Shanghai, China). GelMA was provided by Shuhe Biotechnology (Wenzhou, China). The CCK-8 assay kit, Live/Dead cell viability assay kit, Live/Dead bacterial staining kit, Masson's trichrome staining kit, and DAPI were obtained from Beyotime (Shanghai, China). Mouse fibroblast L929 cells and human corneal epithelial cells (HCEC) were obtained from Wenzhou Medical University (Wenzhou, China). Standard strains of *Staphylococcus aureus* (*S. aureus)*, *Escherichia coli* (*E. coli)*, *Pseudomonas aeruginosa* (*P. aeruginosa)*, and Methicillin-resistant *Staphylococcus aureus* (MRSA) were obtained from the Eye Hospital of Wenzhou Medical University (Wenzhou, China). Dulbecco's modified Eagle's medium (DMEM), fetal bovine serum (FBS), and penicillin–streptomycin solution were purchased from Life Technologies (Gibco, USA). All chemicals and reagents were purchased of analytical grade and used without purification.

### 5.2 Synthesis and characterization of materials

#### 5.2.1 Synthesis of oxidized hyaluronic acid methacryloyl (OHAMA)

Hyaluronic acid (HA, 2.0 g) was dissolved in an ethanol/water solution (10%, w/v), followed by the addition of NaIO_4_ (0.6 g, Aladdin®) to stir at room temperature for 1 h in the dark. Subsequently, the reaction was quenched by adding equimolar ethylene glycol, followed by incubation for an additional 30 min. The resulting suspension was dialyzed against ultrapure water using a dialysis membrane with a molecular weight cut-off MWCO of 7000 Da for 3 days. After lyophilization, the oxidized HA (OHA) powder was obtained. After that, OHA (1.0 g) was dissolved in 100 mL of deionized water, and methacrylate anhydride (1.0 mL, Aladdin®) was added dropwise at 4 °C. The pH value of system was adjusted to 8.0-9.0 using 5 M NaOH and allowed to react for 24 h. The resulting product was then dialyzed against deionized water for 5 days and lyophilized to generate OHAMA powder. The successful synthesis of OHAMA was identified by ^1^H NMR spectroscopy (Bruker 600 MHz, Germany) using D_2_O as the solvent.

#### 5.2.2 Synthesis and characterization of antimicrobial peptide HHC36

HHC36 was synthesized by solid-phase peptide synthesis. Briefly, 2-Cl resin (1.0 g, 1.0 mmol) was swollen in 15 mL dichloromethane (DCM) for 20 min. The first amino acid, (Fmoc-Arg(Pbf)-OH, 617.2 mg, 1.5 mmol), was coupled to the resin using 495 μL N.N-diisopropylethylamine (DIEA) with shaking for 1 h. The resin was then washed with dimethylformamide (DMF) and DCM, and the unreacted sites were capped with a methanol/DIEA/DCM (3:1:16, v/v/v; 15 mL) for 15 min. Fmoc deprotection was performed using ethylenediamine/DMF (1:1, v/v; 15 mL) for 20 min. Peptide chain elongation was carried out through sequential coupling of Fmoc-Arg(Pbf)-OH, Fmoc-Trp(Boc)-OH, Fmoc-Lys(Boc)-OH, Fmoc-Trp(Boc)-OH, Fmoc-Trp(Boc)-OH, Fmoc-Arg(Pbf)-OH, and Fmoc-Lys(Boc)-OH using HBTU (3 mmol) and DIEA (990 μL) with shaking for 2 h, followed by Fmoc deprotection after each coupling step. The final peptide was cleaved from the resin with 15 mL trifluoroacetic acid (TFA) for 30 min. After removal of TFA by evaporation, the residue was precipitated with diethyl ether. The crude peptide was purified by reverse-phase high performance liquid chromatography (RP-HPLC) and lyophilized. The identity and purity of HHC36 peptide was analyzed by LC-MS using an Agilent 1260 Infinity II system (USA).

#### 5.2.3 Preparation of hydrogels

Briefly, LAP was dissolved in phosphate-buffered saline (PBS, pH 7.4) at a concentration of 0.25% (w/v), followed by the dissolution of OHAMA powder giving its final concentrations at 1%, 2%, and 4% (w/v). Similarly, GelMA powder was dissolved into the 0.25% LAP solution to give its final concentration at 20% (w/v). After that, aqueous solutions of OHAMA and GelMA were mixed at equal volumes and vortexed to generate a precursor solution. The resulting samples with different OHAMA concentrations were designated as G_100_OH_5_, G_100_OH_10_, and G_100_OH_20_. The precursor solutions were exposed to visible light irradiation at 405 nm and 10 mw/cm^2^ for 1 min to generate hydrogels. The chemical properties of hydrogels were characterized by Fourier-transform infrared spectroscopy (FTIR; Tensor II, Bruker, Germany) over the spectral range of 500-4000 cm^−1^ (Fig. S3).

#### 5.2.4 Optical properties of hydrogels

The formed GelMA, G_100_OH_5_, G_100_OH_10_, and G_100_OH_20_ hydrogels were first placed on paper sheets printed with the word “eye” and photographed to visually evaluate their transparency. The light transmittance of various hydrogels was then measured using a UV-visible spectrophotometer (Shimadzu UV-1900i, Japan) over a wavelength range of 400-800 nm. Subsequently, the refractive index of various hydrogels was determined using a refractometer (ATAGO NAR-1T, Japan) (n = 3).

#### 5.2.5 Scanning electron microscopy (SEM)

The tested hydrogels were first frozen at −80 °C overnight and subsequently lyophilized. The freeze-dried hydrogels were rapidly frozen in liquid nitrogen and subjected to cryogenic brittle fracture to obtain cross-sectional surfaces. The resulting sections were mounted onto sample stubs, sputter-coated with gold, and observed using scanning electron microscopy (SEM; HITACHI SU8010, Japan).

#### 5.2.6 Contact angle measurement

Water contact angles of various hydrogels were measured using a contact angle goniometer (DataPhysics OCA 25, Germany). Briefly, a 5-μL droplet of deionized water was automatically dispensed onto the sample surface, and its dynamic evolution was recorded with the instrument-integrated camera. Contact angles were automatically calculated using the instrument software (n = 4).

#### 5.2.7 Swelling ratio

The tested hydrogels were weighed to determine their initial weight (W_0_). All samples were then immersed into phosphate-buffered saline (PBS, pH 7.4) at 37 °C for a periodical study. At predetermined time points, each swollen sample was removed, and excess surface solution was carefully wiped off before recording its weight (W_t_). The swelling ratio of each sample was calculated using the following equation: Swelling ratio (%) = (W_t_ - W_0_)/W_0_ × 100% (n = 3).

#### 5.2.8 Water content

After gently blotting the surface moisture of the hydrogels with filter paper, the samples were weighed to determine their wet weight (W_0_). The samples were then freeze-dried and weighed again to obtain their dry weight (W_1_). The water content of the hydrogels was calculated using the following equation: Water content (%) = (W_0_ – W_1_)/W_0_ × 100% (n = 3).

#### 5.2.9 *In vitro* enzymatic degradation test

The hydrogels were initially weighed to determine their initial weight (W_0_) and then immersed into phosphate-buffered saline (PBS, pH 7.4) containing 5 U/mL hyaluronidase (Aladdin®, China) and ≥ 5 CDU/mL collagenase (Worthington Biochemical, USA). The samples were incubated at 37 °C for a period of study, and the weight of the hydrogels (W_n_) was measured at predetermined time points. The residual mass percentage of the hydrogels at each degradation time point was calculated using the following equation: Residual mass (%) = W_n_/W_0_ × 100% (n = 3).

#### 5.2.10 Rheological property

Dynamic photorheological measurements were performed using a photo-rheometer (Kinexus Prime, Germany) equipped with an UV lamp. The sample was placed at the center of two parallel plates with a diameter of 20 mm at room temperature, and the gap between the plates was set to 0.5 mm. The storage modulus (G′) and loss modulus (G″) as functions of time were recorded at a constant angular frequency of 100 rad/s and a strain of 1.0%.

#### 5.2.11 Burst pressure test

A full-thickness circular defect was created at the center of each porcine cornea using a 3 mm trephine. The cornea was then mounted in a custom-built burst pressure testing device and secured between two clamping plates, each containing a 10 mm-diameter central aperture to fully expose the defect area. Subsequently, 50 μL of hydrogel precursor solution was applied dropwise to the defect and rapidly crosslinked under visible-light irradiation. Air pressure was then gradually applied to the system, and the maximum burst pressure was recorded using a wireless pressure sensor (n = 4).

#### 5.2.12 Mechanical tests

For the compression test, hydrogel samples with a diameter of 8 mm and a height of 5 mm were compressed at a rate of 5 mm/min using a universal testing machine (Instron, USA). The compression curves and elastic modulus were recorded. For the adhesive strength test, fresh porcine skin was cut into 1 cm × 1 cm pieces and attached to glass slides. Hydrogel precursor solution (50 μL) was applied between two pieces of porcine skin, which were then pressed together and irradiated with visible light at 405 nm for 1 min. Tensile loading was applied at a rate of 1 mm/min, and the ultimate adhesive strength at the point of separation was recorded.

#### 5.2.13 *In vitro* release profile of HHC36

To determine the *in vitro* release profiles of HHC36 from different hydrogels, a standard curve was first established using UV-visible spectroscopy (Shimadzu UV-1900i, Japan). Briefly, HHC36-loaded hydrogels containing 2 mg/mL HHC36 were fabricated and immersed in 1 mL of phosphate-buffered saline (PBS, pH 7.4) or PBS supplemented with 5 U/mL hyaluronidase (Aladdin®, China) and ≥ 5 CDU/mL collagenase (Worthington Biochemical, USA) at 37 °C to evaluate *in vitro* release. At predetermined time points, 0.5 mL aliquots of the release medium were withdrawn, and the released HHC36 was quantified by UV–visible spectroscopy at a wavelength of 280 nm. After each sampling, an equal volume of fresh release medium was added to maintain a constant release volume.

### 5.3 *In vitro* cell experiments

#### 5.3.1 CCK-8 assay

The *in vitro* cytotoxicity of various hydrogels (G_100_OH_5_, G_100_OH_10_, and G_100_OH_20_ hydrogel) was evaluated using L929 and HCEC cells by the CCK-8 assay. HCEC cells were cultured in DMEM/F12 supplemented with 10% FBS and 1% penicillin-streptomycin, while L929 were cultured in DMEM containing 10% FBS and 1% penicillin-streptomycin. L929 and HCEC cells were seeded in 96-well plates at a density of 2000 cells/well. Sterile hydrogel extracts were prepared by first washing and soaking the hydrogels in PBS for 4 h to thoroughly remove residual LAP. The hydrogels were then immersed in complete culture medium and incubated at 37 °C for 48 h using a mass-to-volume ratio of 0.1 g/mL in accordance with international standards. The resulting extracts were collected and filtered through a 0.22 μm sterile membrane filter prior to use in cell experiments. Subsequently, cells were cultured with the sterile hydrogel extracts for 1, 3, and 5 days at 37 °C in a humidified incubator containing 5% CO_2_. Cell proliferation was quantitatively analyzed using a CCK-8 assay kit (Beyotime, China). The absorbance of samples was measured at 450 nm using a microplate reader (Molecular Devices SpectraMax M5, USA) (n = 6).

#### 5.3.2 Two-dimensional (2D) cell culture

Briefly, 100 μL of sterile hydrogel precursor solution was added to each well of a 96-well plate and then gelled by exposure to visible light at 405 nm for 1 min. After that, L929 cells were seeded onto the hydrogel surfaces at a density of 2000 cells/well and the cell viability was assessed by a Live/Dead assay kit (Beyotime, China) on day 1, 3, and 5. Live and dead cells were visualized by green and red fluorescence, respectively, using an inverted fluorescence microscope (Leica DMi8, Germany).

#### 5.3.3 Three-dimensional (3D) cell culture

Briefly, 100 μL of the cell-laden precursor solution (containing 2 × 10^5^ L929 cells) was added to each well of a 96-well plate and then gelled by exposure to visible light at 405 nm for 1 min. Cell viability within the hydrogels was evaluated using a Live/Dead assay kit on day 1, 3, and 5. The samples were imaged in 3D reconstruction mode using a confocal laser scanning microscope (LSM 880, Carl Zeiss, Germany).

#### 5.3.4 Cell migration assay

Briefly, HCECs were seeded in 6-well plates at a density of 1 × 10^6^ cells per well and cultured until approximately 90% confluence was reached. Subsequently, a straight scratch was created across the cell monolayer using a sterile 200 μL pipette tip. After rinsing with phosphate-buffered saline (PBS, pH 7.4) triplicate, 2 mL of sterile hydrogel extract prepared in low-serum medium was added to each well, with low-serum medium alone serving as the negative control. Cell migration was imaged at 0, 24, and 36 h using an inverted fluorescence microscope (DMI8, Leica, Germany), and the images were analyzed with ImageJ software.

### 5.4 *In vitro* antimicrobial activity

#### 5.4.1 IC_50_ assay of HHC36

Standard strains of *S. aureus*, *E. coli*, *P. aeruginosa*, and MRSA were obtained from the Eye Hospital of Wenzhou Medical University. The strains were streak-inoculated onto blood agar plates and incubated at 37 °C for 24 h. Bacterial suspensions were then adjusted to a 0.5 McFarland standard. Subsequently, 120 μL of each bacterial suspension was mixed with 12 mL of antimicrobial susceptibility testing medium, followed by the addition of 100 μL of the mixture to each well of a 96-well plate. HHC36 peptide was added at gradient concentrations. Bacteria without peptide served as the positive control, while medium alone served as the negative control. After incubation at 37 °C for 24 h, the absorbance (A) at 600 nm was measured using a microplate reader. The antibacterial rate was calculated using the following equation: Antibacterial rate (%) = [A(positive) − A(test)]/[A(positive) − A(negative)] × 100%. All experiments were performed in triplicate (n = 4).

#### 5.4.2 *In vitro* antibacterial activity of HHC36@Gel

Bacterial suspensions of MRSA and *P. aeruginosa* at a concentration of 1 × 10^6^ CFU/mL were incubated with blank hydrogel (G_100_OH_10_ hydrogel), HHC36@Gel^1^ (1 mg/mL HHC36), or HHC36@Gel^2^ (2 mg/mL HHC36) for 24 h, with wells without hydrogels serving as controls. Subsequently, the bacterial suspensions from each group were serially diluted, spread onto agar plates, and incubated 24 h for colony counting assay. For Live/Dead staining, bacterial suspensions from each group were centrifuged at 10,000 × g for 5 min, washed three times with saline, and stained using a Live/Dead bacterial staining kit (Beyotime, China). The stained bacteria were then visualized under a fluorescence microscope (n = 5).

#### 5.4.3 Encapsulation efficiency of HHC36 in hydrogels

HHC36-loaded hydrogel samples (0.5 mL per sample) were prepared at an initial HHC36 concentration of 2 mg/mL (n = 4 for each group). Each hydrogel sample was gently washed with PBS four times for 5 min per wash, and all washing solutions were collected. The concentration of unencapsulated HHC36 in the collected washing solutions was determined using ultraviolet spectrophotometry. The amount of HHC36 encapsulated within the hydrogel was calculated by subtracting the amount of unencapsulated HHC36 from the initial amount of HHC36 added during hydrogel preparation. The encapsulation efficiency (EE) was calculated using the following equation: EE% = [W(total) - W(free)]/W(total) × 100%, where W(total) represents the initial amount of HHC36 added during hydrogel preparation, and W(free) represents the total amount of unencapsulated HHC36 recovered from the washing solutions.

#### 5.4.4 Bacterial membrane integrity

Briefly, 1 mL bacterial suspensions (1 × 10^6^ CFU/mL) were incubated with 1 mL hydrogels for 24 h. After centrifugation at 4000 rpm for 10 min at 4 °C, the samples were washed three times and fixed overnight in 2.5% glutaraldehyde. The samples were then washed three times with ultrapure water by centrifugation at 4000 rpm for 4 min each, followed by dehydration in a graded ethanol series: 50-90% for 15 min each, and 100% ethanol for 15 min twice. After air-drying on silicon wafers, the samples were sputter-coated with gold and observed using scanning electron microscopy (SEM).

#### 5.4.5 Biofilm inhibition

Briefly, 100 μL bacterial suspensions (1 × 10^6^ CFU/mL) incubated with 100 μL hydrogels at 37 °C for 48 h. After that, the wells from each group were then washed with PBS, dried at 37 °C for 30 min, and fixed with 4% paraformaldehyde for 30 min. After washing and air-drying, the biofilms were stained with 0.2% crystal violet for 30 min. The wells were subsequently washed, dried, and photographed. The bound crystal violet was dissolved in anhydrous ethanol for 15 min, and the absorbance was measured at 570 nm. All experiments were performed in triplicate (n = 4).

#### 5.4.6 *In vitro* cytotoxicity of HHC36

HCEC and L929 cells in the logarithmic growth phase were seeded into 96-well plates at a density of 2 × 10^3^ cells/well in 100 μL of culture medium and incubated at 37 °C for 24 h. Lyophilized HHC36 powder was dissolved in PBS to prepare an 800 μg/mL stock solution, which was subsequently diluted with complete culture medium to obtain HHC36 solutions at concentrations of 200, 100, 50, 25, 12.5, and 6.25 μg/mL. Subsequently, 100 μL of HHC36 solution at each concentration was added to the corresponding wells. On days 1, 3, and 5, cell viability was assessed using the MTT assay. Briefly, 20 μL of MTT solution (5 mg/mL) was added to each well, followed by incubation in the dark for 2 h. After incubation, the supernatant was carefully aspirated, and 150 μL of dimethyl sulfoxide (DMSO) was added to each well to dissolve the purple formazan crystals produced by metabolically active cells. The plates were then shaken for 15 min in the dark to ensure complete dissolution of the crystals. Absorbance was measured at 490 nm using a microplate reader, and cell viability was calculated using the following equation: Cell viability (%) = A(treated)/A(untreated) × 100%, where A(treated) and A(untreated) represent the absorbance values of HHC36-treated and untreated control cells, respectively.

### 5.5 Animal experiments

Healthy adult male New Zealand White rabbits weighing 2.5-3.0 kg were obtained from Yuhang Kelian Rabbit Industry Co., Ltd (Hangzhou, China). Specific anesthetic used and dosages are as follows: Rabbits were euthanized by intravenous injection of Barbital Sodium (Sigma-Aldrich, USA, 100 mg/kg). Anesthesia was performed by intraperitoneal injection of Sumianxin Ⅱ (Shengxin, China, 0.3 mL per rabbit) and Barbital Sodium (20 mg/mL, 2 mL per rabbit) prior to the establishment of animal model and optical imaging, and topical anesthesia was applied using proparacaine (5 mg/mL, 0.05 mL per drop) eye drops.

#### 5.5.1 Rabbit deep corneal stromal defect model and medication

All surgical instruments were sterilized by autoclaving before use. To establish the corneal stromal defect model, a deep stromal defect was created in the central cornea using a 3 mm trephine. Subsequently, 2.5 μL of G_100_OH_10_ hydrogel was applied to the defect area in the hydrogel group, whereas the model group received no repair treatment (n = 8 for each group). Postoperatively, levofloxacin eye gel was administered three times daily for 7 consecutive days to prevent bacterial infection.

#### 5.5.2 Rabbit deep corneal stromal defect model complicated with *P. aeruginosa* infection and medication

Rabbit deep corneal stromal defects were established as described above. *P. aeruginosa* suspension (5 μL) at a concentration of 1 × 10^8^ CFU/mL was applied to the defect area for 1 min, after which excess bacteria were gently removed using a sterile cotton swab. The infected defects were then treated with HHC36@Gel^2^ (2 mg/mL HHC36). Plain hydrogel (G_100_OH_10_ hydrogel) was used as the negative control, and plain hydrogel (G_100_OH_10_ hydrogel) combined with levofloxacin eye drops (50 μL; 4.88 mg/mL) administered three times daily served as the positive control (n = 8 for each group).

#### 5.5.3 Slit lamp, AS-OCT, and specular microscopy observations

Slit-lamp examination was performed using a slit-lamp microscope system (Kanghua Ruiming Technology, China) to evaluate therapeutic efficacy. Corneal transparency and epithelial migration were assessed under 10× magnification using bright-field illumination and fluorescein sodium staining. Cross-sectional images of the cornea were acquired using anterior segment optical coherence tomography (AS-OCT; Heidelberg Spectralis OCT, Germany) to evaluate corneal curvature, hydrogel degradation, and hyperreflective deposits in the corneal stroma. Specular microscopy was performed using a specular microscope (TOPCON SP-1P, Japan) to assess corneal endothelial morphology and density.

#### 5.5.4 Histological analysis

Rabbits from each group were sacrificed at predetermined time points. The eyeballs were enucleated and fixed overnight in FAA solution (formaldehyde/acetic acid/70% ethanol = 5:5:90, v/v/v), dehydrated through a graded ethanol series, embedded in paraffin, and sectioned (∼5 μm thickness) for hematoxylin and eosin (H&E) and Masson's trichrome staining (Solarbio, China).

For immunostaining assay, eyeballs were fixed in 4% paraformaldehyde for 4 h, embedded in O.C.T compound (Sakura Finetek, Japan) using liquid nitrogen, and sectioned at 10 μm thickness. The sections were incubated overnight at 4 °C with the following primary antibodies: CK3 (1:100, Abcam, UK), ZO-1 (1:50, Invitrogen, USA), TGF-β (1:200, Gene Tex, USA), α-SMA (1:200, Invitrogen, USA), vimentin (1:200, Invitrogen, USA), TNF-α (1:200, NeoBiotechnologies, USA), IL-1β (1:200, Proteintech, China), CD68 (1:200, Bio-Rad, USA), CD11b (1:200, Zen-BioScience, China), and MMP9 (1:200, Abcam, UK). After washing with PBS, the sections were incubated with the corresponding secondary antibodies (Invitrogen, USA) at room temperature for 2 h in the dark, counterstained with DAPI (Beyotime, China), and imaged using a confocal fluorescence microscope (ZEISS LSM 880, Germany). Fluorescence intensity was semi-quantified using ImageJ software.

#### 5.5.5 Corneal nerve staining

Corneas from each group were fixed in 4% paraformaldehyde at 4 °C for 4 h, permeabilized with 1% Triton X-100 in PBS for 1 h, and blocked with 1% BSA in PBS for 1 h. The tissues were then incubated overnight at 4 °C with an anti-β3-tubulin antibody (BioLegend, USA), followed by rinsing with PBS triplicate. The corneas were cut into a four-leaf clover shape, flattened onto glass slides, mounted with antifade mounting medium, and sealed. Images were acquired using a confocal fluorescence microscope (ZEISS LSM 880, Germany).

#### 5.5.6 Transmission electron microscopy (TEM) observation of corneal tissue

Corneas from each group were fixed in 2.5% glutaraldehyde at 4 °C, washed with PBS, post-fixed in 1% osmium tetroxide for 1 h, and stained with uranyl acetate for 2 h. After gradient dehydration in acetone, the samples were infiltrated overnight with a mixture of acetone and Epon resin, embedded in pure Epon, and polymerized. Semithin sections were first prepared for target localization, followed by ultrathin sectioning for observation using TEM (HITACHI H-7500, Japan).

#### 5.5.7 Biocompatibility of hydrogel implantation

Rabbits were anesthetized and euthanized on day 90 post-surgery. Major organs, including the heart, liver, spleen, lung, and kidney, were collected from normal rabbits and hydrogel-implanted (G_100_OH_10_ hydrogel) rabbits for H&E staining to evaluate the biocompatibility of hydrogel implantation.

#### 5.5.8 Corneal bacterial colony counting

To evaluate the therapeutic efficacy of HHC36@Gel^2^, corneal tissues from each group were collected from each group on day 7 post-surgery. The tissues were placed in 1 mL of sterile PBS and thoroughly homogenized using a tissue homogenizer. The supernatant was then evenly spread onto agar plates and incubated overnight at 37 °C. Viable bacterial counts were determined by counting colony-forming units (CFUs) on the agar plates (n = 4).

### 5.6 Statistical analysis

All statistical analyses for the above experiments were performed using GraphPad Prism 8.00 and Origin 7.5 software. Quantitative data are presented as the mean ± standard deviation (SD). Statistical comparisons were performed using analysis of variance (ANOVA) or Student's *t*-test, as appropriate. A *P*-value of < 0.05 was considered statistically significant.

## CRediT authorship contribution statement

**Wei Wu:** Writing – original draft, Visualization, Validation, Methodology, Investigation, Formal analysis, Data curation, Conceptualization. **Shuo Huai:** Visualization, Investigation, Formal analysis, Data curation, Conceptualization. **Chenhao Li:** Methodology, Formal analysis, Data curation. **Ling Wang:** Investigation. **Siqi Xie:** Validation. **Lei Lei:** Investigation. **Xiaoying Liu:** Validation. **Jiaqing Wang:** Writing – review & editing, Writing – original draft, Supervision, Resources, Project administration, Funding acquisition, Conceptualization. **Xingyi Li:** Writing – review & editing, Writing – original draft, Supervision, Resources, Project administration, Funding acquisition, Conceptualization.

## Data availability statement

The data that support the findings of this study are available from the corresponding author upon reasonable request.

## Ethics approval and consent to participate

All animal experiments were conducted in accordance with the guidelines for the care and use of laboratory animals. The study was approved by the Animal Care and Use Committee of Wenzhou Medical University (Approval No. YSG26062226). All procedures followed the 3R principles. This study does not involve human participants, so informed consent is not applicable.

## Declaration of competing interest

The authors declare that they have no known competing financial interests or personal relationships that could have appeared to influence the work reported in this paper.

## Appendix A Supplementary data

Synthetic route, GPC spectrum, ^1^H NMR spectrum, LC-MS spectrum, FTIR spectrum, OD_600_ results of hydrogel against MRSA and *P. aeruginosa*, immunofluorescence staining images of α-SMA and vimentin, and H&E staining images of major organs.
