# Supplementary material for: An optically and biomechanically corneal tissue-matched dual-crosslinkable hydrogel for cell-free remodeling of the corneal stroma
Source: Bioact Mater. 2026 Oct 31;70:23–43. doi: 10.1016/j.bioactmat.2026.10.032 (PMC13743903; doi:10.1016/j.bioactmat.2026.10.032)
Supplement: Supplementary file 1 [file mmc1.docx]

Supporting information for

An optically and biomechanically corneal tissue-matched dual-crosslinkable hydrogel for cell-free remodeling of the corneal stroma

**Scheme S1.** Synthetic route of OHAMA.

**Scheme S2.** Synthetic route of HHC36.

**Figure S1.** ^1^H NMR spectrum of HA and OHAMA in D_2_O.

**Figure S2.** GPC (a) spectrum, and (b) results of OHAMA.

**Figure S3.** (a) FTIR spectrum and (b) Raman spectrum of HA, OHAMA, GelMA, G_100_OH_10_ hydrogel precursor mixture, and G_100_OH_10_ hydrogel.

**Figure S4.** (a) Quantification of the pore size and (b) porosity of various hydrogels.

**Figure S5.** (a) Photograph of the corneal burst pressure testing device. (b) Burst pressure of ex vivo porcine corneal defect models treated with different hydrogels.

**Figure S6.** Quantitative analysis of (a) cell viability and (b) live cell counts in 2D cultures, and (c) cell viability and (d) live cell counts in 3D cultures within different hydrogel formulations on days 1, 3, and 5.

**Figure S7.** Representative AS-OCT images of rabbit corneas treated with G_100_OH_10_ at different times. **Note:** The images of Day 0, Day 7, Day 30, and Day 90 are identical to those presented in Figure 3 of the main text.

**Figure S8.** (a) Representative bright-field (BF), slit-lamp (SL), and fluorescein staining (FL) images of rabbit eyes from different groups. (b) Representative AS-OCT images of rabbit corneas from different groups. (c) H&E staining images of rabbit corneal tissues from different groups at day 30. **Note:** The images of the Normal, Model, and G_100_OH_10_ groups are identical to those presented in Figure 3 of the main text.

**Figure S9.** LC-MS spectrum of HHC36.

**Figure S10.** Fluorescence microscopy images showing the distribution of Rhodamine B with different hydrogels.

**Figure S11.** (a) Refractive index, (b) swelling ratio, (c) compressive stress-strain curves, and (d) elastic modulus of G_100_OH_10_ before and after HHC36 incorporation. **Note:** All results of G_100_OH_10_ are identical to those presented in Figure 1 of the main text.

**Figure S12.** Half-maximal inhibitory concentration (IC_50_) of HHC36 against MRSA and *P. aeruginosa* after incubation in PBS, simulated tear fluid (STF), and STF containing 0.01% lysozyme for (a, b) 2 h, (c, d) 24 h, (e, f) one week, (g, h) two weeks, and (i, j) one month, respectively.

**Figure S13.** (a) LC and (b) MS spectra of HHC36 after storage for three months. Half-maximal inhibitory concentration (IC_50_) of HHC36 against (c) MRSA and (d) *P. aeruginosa* after storage for three months.

**Figure S14.** Quantitative results of optical density (OD) values using (a) MRSA and (b) *P. aeruginosa*.

**Figure S15.** Cell viability of (a) L929 and (b) HCEC treated with HHC36 at different concentrations for 1, 3, and 5 days.

**Figure S16.** (a) Immunofluorescence staining image of α-SMA, and vimentin in cornea on day 30. Quantitative fluorescence of (b) α-SMA, and (c) vimentin in cornea on day 30.

**Figure S17.** H&E staining of major organs (heart, liver, spleen, lung, and kidney) from Normal rabbit and G_100_OH_10_ hydrogel-implanted rabbit on day 90.
